# Anxiety associated with perceived uncontrollable stress enhances expectations of environmental volatility and impairs reward learning

**DOI:** 10.1038/s41598-023-45179-z

**Published:** 2023-10-27

**Authors:** Marc Guitart-Masip, Amy Walsh, Peter Dayan, Andreas Olsson

**Affiliations:** 1 Department of Neurobiology, Care Sciences and Society, Karolinska Institutet, Aging Research Centre, Stockholm, Sweden; 2 Center for Psychiatry Research, Region Stockholm, Stockholm, Sweden; 3 Karolinska Institutet, Center for Cognitive and Computational Neuropsychiatry (CCNP), Stockholm, Sweden; 4 Emotion Lab, Department of Clinical Neuroscience, Karolinska Institutet, Stockholm, Sweden; 5 Max Planck Institute for Biological Cybernetics, Tübingen, Germany; 6 University of Tübingen, Tübingen, Germany

**Keywords:** Psychology, Human behaviour

## Abstract

Unavoidable stress can lead to perceived lack of control and learned helplessness, a risk factor for depression. Avoiding punishment and gaining rewards involve updating the values of actions based on experience. Such updating is however useful only if action values are sufficiently stable, something that a lack of control may impair. We examined whether self-reported stress uncontrollability during the first wave of the COVID-19 pandemic predicted impaired reward-learning. In a preregistered study during the first-wave of the COVID-19 pandemic, we used self-reported measures of depression, anxiety, uncontrollable stress, and COVID-19 risk from 427 online participants to predict performance in a three-armed-bandit probabilistic reward learning task. As hypothesised, uncontrollable stress predicted impaired learning, and a greater proportion of probabilistic errors following negative feedback for correct choices, an effect mediated by state anxiety. A parameter from the best-fitting hidden Markov model that estimates expected beliefs that the identity of the optimal choice will shift across images, mediated effects of state anxiety on probabilistic errors and learning deficits. Our findings show that following uncontrollable stress, anxiety promotes an overly volatile representation of the reward-structure of uncertain environments, impairing reward attainment, which is a potential path to anhedonia in depression.

## Introduction

To obtain rewards successfully from complex, ever-changing environments, people flexibly learn to adapt behaviour based on prior experience^1–3^. Better than expected outcomes elicit positive prediction errors, while worse than expected outcomes elicit negative prediction errors, thus updating estimated action values to support optimal choices^4,5^. Stress can interfere with this learning process, damaging reward maximization^6^ and impairing avoidance of aversive outcomes^7^. For example, anticipating a shock impairs performance in probabilistic reward learning tasks^8–10^. It is, however, unknown if the perceived uncontrollability of stress (i.e., unavoidable through one’s actions; a key form of helplessness^11^) determines the extent of these cognitive consequences^12,13^.

When actions result in desired outcomes, a subjective sense of controllability or agency arises that leads to exploration and goal-directed action^12,14,15^. It is thought that expectations regarding controllability are determined through generalization from experiences in similar contexts^16–18^. When actions do not lead to desired consequences, a sense of uncontrollability leads to reflexive, passive behaviour and learned helplessness (a failure to attempt to avoid *controllable* stressors in new contexts^13,15,16^). Given the contribution of stress^19–21^ and uncontrollability^22^ to aspects of depression, learned helplessness is a widely used model of depression^13,21,23^.

It is well established that learned helplessness disturbs the normal course of action learning^13,21^. However, there are many potential computational mechanisms that may give rise to impaired learning. Some of these have been systematically studied in learned helplessness and anhedonia, namely issues with reward processing, and biases or deficits in the learned associations between stimuli and/or actions and rewards^21,24–29^. Another potential source of the observed deficits in action learning may be an inability to adapt behaviour appropriately when changing reward contingencies induce second-order uncertainty^30^. In these circumstances, problems may arise for learning if subjects believe they cannot control whether environmental relationships are long lasting. In fact, one of the most robust findings in both depression and state and trait anxiety is a disruption to reward learning coming from dysfunctional behavioural adjustments to the rate of change in reward contingencies^24,31–36^. However, the ability to adapt to changing reward contingencies in learned helplessness has not previously been studied.

**Figure 1:**
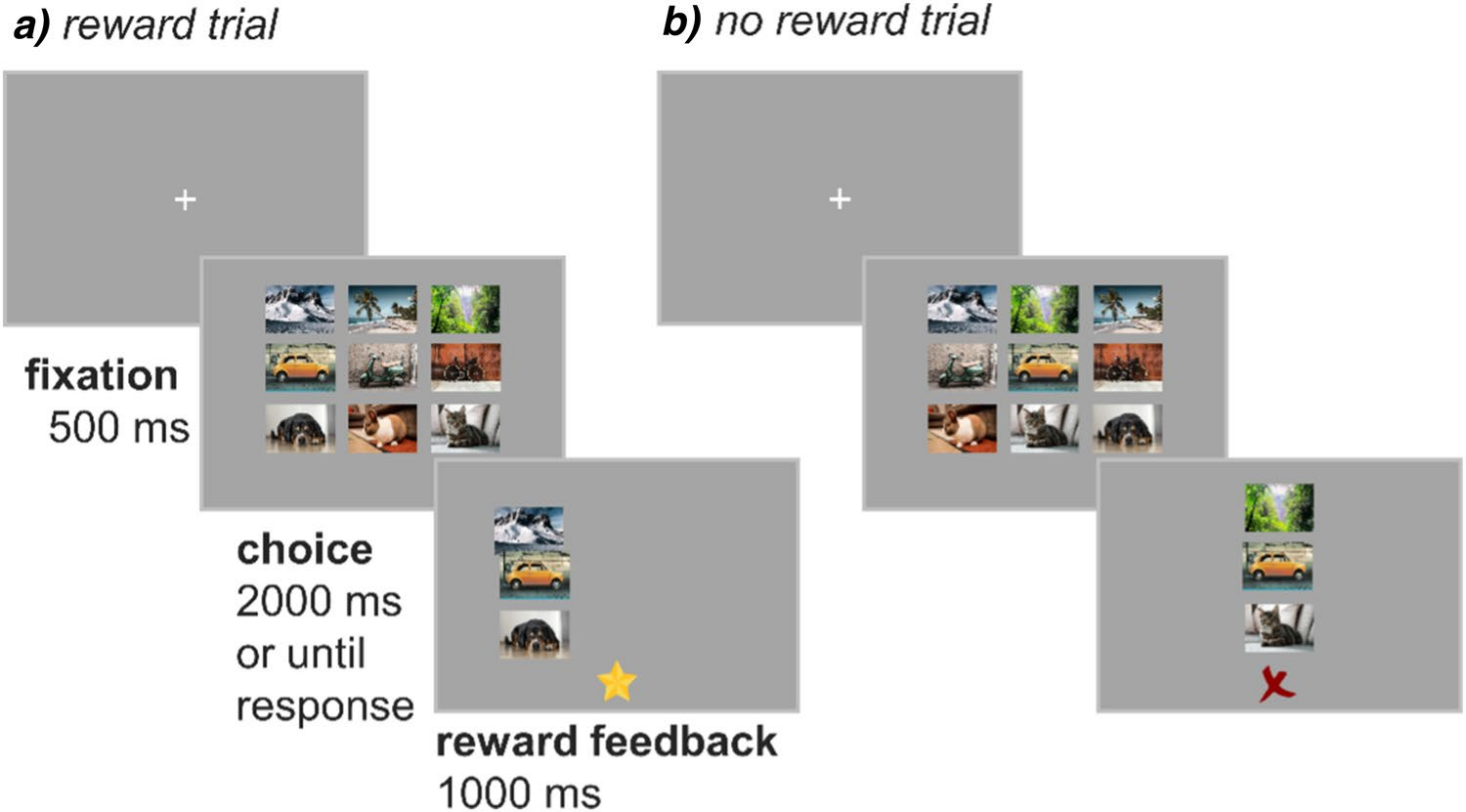
A trial diagram showing a rewarded trial (**a**), and an no reward trial (**b**). Participants saw a fixation cross for 500 ms, followed by the image stimuli until a response was made, or for 2000 ms if they failed to respond in time. Participants selected one of the three vertical composite stimuli (comprising one landscape, one animal, and one mode of transport) on each trial using the arrow keys (left, down, right). Images in each horizontal row were from the same class (landscape; mode of transport; animal) but were randomly shuffled within row on each trial. On any given trial, one of the images (e.g., a cat) was the target. Participants had to learn by accumulating evidence from their composite choices. Following participants choices, reward feedback was presented along with the chosen composite stimulus: a star on reward trials, and a cross on no reward trials. If no response was made, an image of a clock was shown to remind participants to respond more quickly. Participants first completed the signalled task in which they were informed that the target had changed at the start of each new game of 25 trials (5 games in total). Choosing the composite stimulus with the target had a reward probability of 0.75 whereas non-target composites had a reward probability of 0.25. Participants then completed the reversal task, which comprised one continuous game of 125 trials in which the target changed without warning every 20–30 trials. Participants were informed about the existence of the reversals. As the reversal task was more difficult than the signalled task, we increased reward probability upon choosing the target to 0.8 (0.2 for non-targets).

In this preregistered study, we ask whether uncontrollable stress is associated with impaired reward learning and examine possible computational mechanisms by which this might arise. The COVID-19 pandemic presented a stressful context, with naturally varying subjective responses across individuals^37,38^. During the first-wave of the COVID-19 pandemic in April 2020, 427 online participants self-reported levels of perceived uncontrollable stress and lack of self-efficacy^39^, depressive symptoms^40^, state and trait anxiety^41,42^, and perceived risk of COVID-19^43^. Participants also performed two reward learning tasks (Fig. 1) adapted from Leong et al^44^ that differed in the level of second-order uncertainty. 49 of the same participants completed an identical session approximately 3 days later.

In both tasks, the goal was to maximise monetary reward. On each trial, participants chose one of three vertical columns, each comprising three images. Images in each horizontal row were from the same class (landscape; mode of transport; animal) but were randomly shuffled within row on each trial. On any given trial, one of the images (e.g., a cat) was the target. Participants had to learn by accumulating evidence from their composite choices. Participants first completed the *signalled task* in which they were informed that the target had changed at the start of each new game of 25 trials (5 games in total). Choosing the composite stimulus with the target had a reward probability of 0.75 whereas non-target composites had a reward probability of 0.25. Participants then completed the *reversal task*, which comprised one continuous game of 125 trials in which the target changed without warning every 20–30 trials. Participants were informed about the existence of the reversals. As the reversal task was more difficult than the signalled task, we increased reward probability upon choosing the target to 0.8 (0.2 for non-targets). Participants were informed about reward probabilities for both tasks. As the learning task is very challenging, we reasoned that it would be easier for participants to learn to perform the task without reversal first, and only afterwards to face the reversal component. We expected that uncontrollable stress would impair learning in both tasks. To examine possible cognitive mechanisms by which uncontrollable stress might impair reward learning, we compared different computational models that were fit to the participants’ choices.

## Results

### Calculating factor scores from the questionnaires

As preregistered, we initially used Exploratory Factor Analysis (EFA) to derive latent factor scores within and across questionnaires (see supplementary methods). However, these EFA scores were not reliable as assessed on the 49 participants who completed the scales twice 3 day apart (ICC^45,46^ ranged between 0.14 and 0.61; see Table S.1). This was surprising as the sum scores for each scale and subscale showed good test–retest reliability (ICC range: 0.75–0.95). As scales are normally validated using factor analysis, using sum scores implies using a model that is different from the validation model^47^. As such, the use of sum scores is not recommended^47^. We deviated from the preregistration and calculated congeneric factor scores, using confirmatory factor analysis to produce weighted scores based on previously established structure of the questionaires^47^. In this way, the scores we used are closer to the latent construct that the scales are meant to measure. See “Materials and methods” for details. We also determined whether each scale should be subset into previously established subscales.

Model fit comparison (Table S.2) of the one-factor (full scale) and two-factor (subscales) models determined the following factor structure: Uncontrollable Stress; Lack of Self-efficacy; Depression; Perceived Likelihood of COVID-19 Risk; Perceived Severity of COVID-19 Risk; State Anxiety (negatively-framed items), State Anxiety (positively-framed items), Trait Anxiety (negatively-framed items), and Trait Anxiety (positively-framed items). Unlike the EFA factor scores, congeneric scores were test–retest reliable (ICC range: 0.75–0.93; Table S.3).

### Impact of uncontrollable stress and state anxiety on reward learning

Distribution of learning measures are displayed in supplemental Fig. S.1. To examine which factors affected learning, separately for the signalled and reversal tasks, we ran preregistered generalised logistic mixed-models (GLMMs) on accuracy (0, 1), with Trial (centred around 0) as a fixed-effect, and by-subject random intercepts and slopes for Trial, thus allowing for between-subject learning variability. We systematically added Factor Score × Trial interactions as fixed-effects and then as random-effects in separate models for each of the nine factors. Adding random-effect interaction terms did not significantly improve fit of any of the models. We note that in the preregistration we planned to use factor scores from the EFA, but because of low reliability we use factor scores from the confirmatory factor models.

Learning in the signalled task was not modulated by any of the factors (see Table S.6 for GLMMs). Therefore, the signalled task was not considered further.

**Figure 2:**
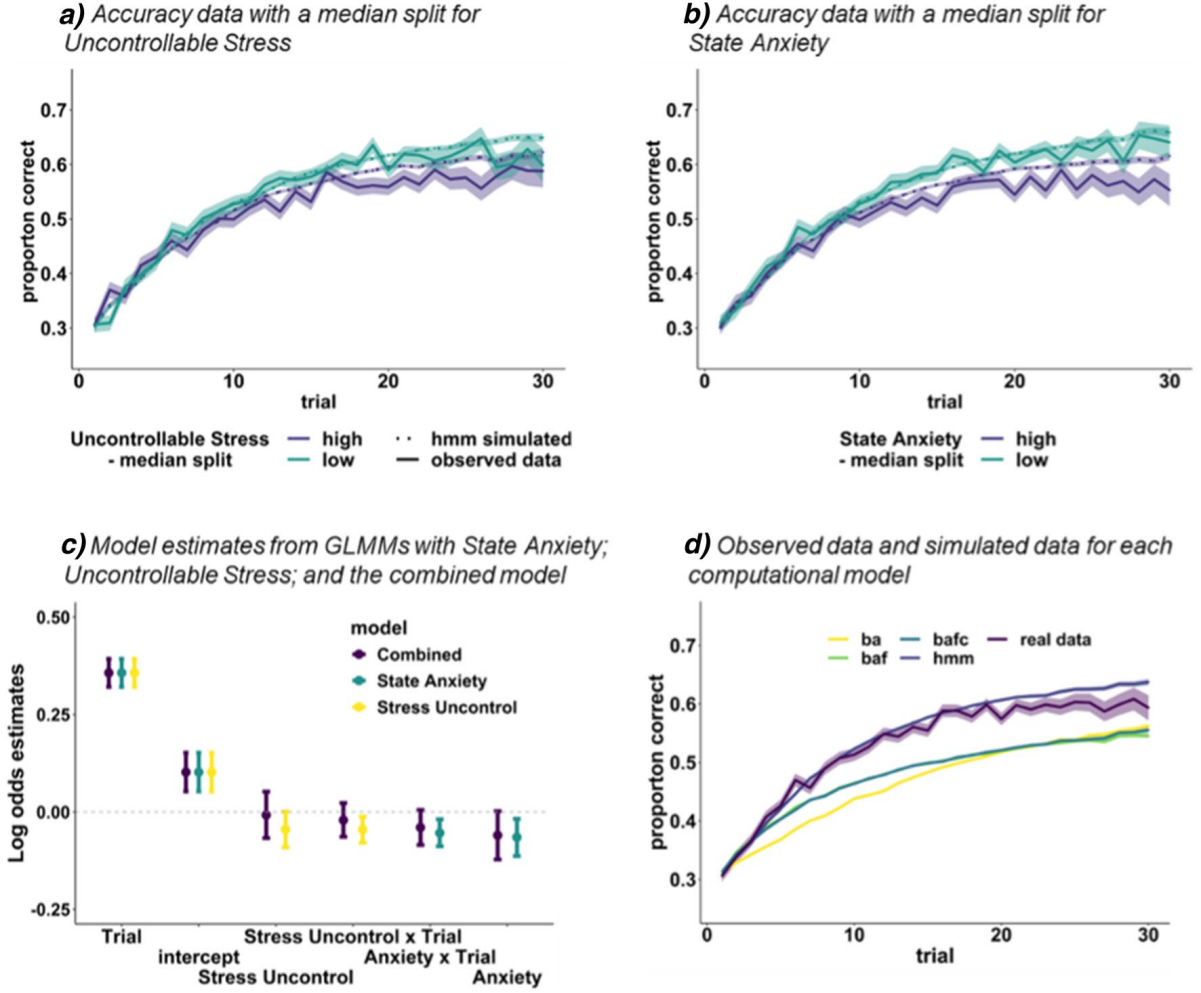
The effects of Uncontrollable Stress and State Anxiety on reward learning (accuracy across trials) in the reversal task (n = 427). (**a**) Shows reward learning collapsed across games with a median split of Uncontrollable Stress scores; and a median split of State Anxiety scores in (**b**). High scorers are in purple and low scorers are in green. These median splits are only for the purpose of visualisation, and we treated the factor scores as continuous variables in analyses. Shaded error bars are standard errors of the mean. Solid lines indicate the observed data, dotted lines indicate simulated accuracy by the winning computational hidden Markov model (HMM, see methods for details). (**c**) shows the estimates (log odds) from the Generalised Logistic Mixed Models (GLMMs) that included the Uncontrollable Stress × Trial interaction alone (yellow), the State Anxiety × Trial interaction alone (green), and the combined model with both interactions (purple). Error bars reflect 95% confidence intervals around the mean estimates. Error bars that cross the dotted horizontal line indicate non-significant effects. For results of the mediation model see Table 3. (**d**) The observed accuracy data (“real data”) and simulated choice data created by the various reinforcement learning models (RLMs), and the hidden Markov Model (HMM). ba = beta-alpha; baf = beta-alpha-forget; bafc = beta-alpha-forget-confidence; hmm = hidden Markov model with 3 free parameters. The simulations were done by sampling each individual subject's set of parameters 100 times (see “Materials and methods” for details). The HMM is clearly the best fitting model to participants’ choices, although we note that the simulated data deviates slightly from the observed data from trial 20–30.

The reversal task GLMM results are presented in Table 1. Importantly, as predicted in the preregistration, an Uncontrollable Stress × Trial interaction supported our key hypothesis that perceived lack of stress controllability is associated with impaired reward learning. As positively-framed State Anxiety had no effects on learning, we refer to negatively-framed State Anxiety simply as State Anxiety. State Anxiety significantly predicted lower accuracy and impaired learning (a State Anxiety × Trial interaction). With Holm correction for multiple comparisons^48^ only the State Anxiety × Trial interaction remained significant (Table 1). These significant effects were not dependent on using congeneric scores and were replicated when using the respective summed score (see supplemental table S.8). Although the Uncontrollable Stress x Trial interaction did not survive the Holm correction, it supported our key preregistered and theoretically driven prediction. Indeed, the effects of other factors were exploratory in the preregistration. See Fig. 2 for a depiction of effects of Uncontrollable Stress and State Anxiety on learning, with a median split of participants for visualisation purposes only (see supplementary Fig. S2 for a distribution of the scores for state and trait anxiety as well as the perceived stress scale along with stablished cutoffs).

**Table 1:** Generalised logistic mixed model (GLMM) results for the reversal task: results from the nine separate models with Factor Score × Trial interactions.

Model	Fixed effects	Log odds	se	z value	*p* value	CI lower	CI higher
**Uncontroll-able stress**	Main effect	− 0.045	0.023	− 1.905	0.057	− 0.091	0.001
	**Uncontrollable stress × Trial**	− **0.045**	**0.017**	− **2.655**	0.008*	− **0.079**	− **0.012**
**State anxiety neg-framed**	**Main effect**	− **0.065**	**0.024**	− **2.685**	0.007*	− **0.113**	− **0.018**
	**State anxiety × Trial**	− **0.054**	**0.018**	− **3.047**	0.002**	− **0.088**	− **0.019**
Lack of self- efficacy	Main effect	− 0.007	0.023	− 0.287	0.774	− 0.051	0.038
	Lack of self- efficacy × Trial	0.004	0.017	0.267	0.789	− 0.028	0.037
Depression	Main effect	− 0.030	0.024	− 1.240	0.215	− 0.078	0.017
	Depression × Trial	− 0.006	0.018	− 0.356	0.722	− 0.041	0.028
Likely COVID-19 risk	Main effect	− 0.022	0.024	− 0.905	0.365	− 0.069	0.025
	Likely COVID-19 Risk × Trial	0.003	0.018	0.181	0.856	− 0.031	0.038
Severity COVID-19 risk	Main effect	− 0.007	0.022	− 0.338	0.735	− 0.050	0.035
	Severity COVID-19 Risk × Trial	− 0.012	0.016	− 0.761	0.446	− 0.043	0.019
Trait anxiety neg-framed	Main effect	− 0.044	0.024	− 1.817	0.069	− 0.091	0.003
	Trait anxiety neg-framed × Trial	− 0.016	0.018	− 0.879	0.379	− 0.050	0.019
State anxiety pos-framed	Main effect	− 0.045	0.025	− 1.834	0.067	− 0.093	0.003
	State anxiety pos-framed × Trial	− 0.033	0.018	− 1.832	0.067	− 0.068	0.002
Trait anxiety pos-framed	Main effect	− 0.020	0.024	− 0.801	0.423	− 0.067	0.028
	Trait anxiety pos-framed × Trial	0.002	0.018	0.128	0.898	− 0.033	0.037

Confidence intervals are 95%. se is the standard error of the log odds estimate. Significant fixed-effects and interactions are shown in bold. Log odds estimates can be transformed into odds ratios by exponentiating the value. Across these nine models, the coefficients for the intercept ranged from: log odds = 0.102, se = 0.025, z = 4.023–4.056, p < 0.001, 95% CI = 0.053–0.152; and for the main effect of Trial: log odds = 0.357, se = 0.018–0.019, z = 19.232–19.461, p < 0.001, 95% CI = 0.321–0.394. With a Holm correction for multiple comparisons (Holm, 1979) only the negatively-framed State Anxiety x Trial interaction remained significant. Although the Uncontrollable Stress × Trial interaction did not survive the Holm correction, it supported our key preregistered theoretically-driven prediction; effects of all other factors were relatively exploratory. *One asterisk indicates a significant effect before the Holm correction; **two asterisks indicate a Holm-corrected significant effect. A Holm correction is more powerful than a Bonferroni correction, is valid under the same assumptions, and controls the family-wise error rate. To perform the Holm correction, p-values are ordered from smallest to largest. A p-value is significant when pk < α/(m + 1−k), where α is the alpha level; m is the number of p-values (18 in this case); and k is the p-value ranking. For all three significant effects, the adjusted alpha threshold rounded to 0.003.

Uncontrollable Stress × Trial and State Anxiety × Trial were entered into a combined GLMM as fixed-effects (Fig. 2; Table S.5). Variance inflation factors were < 2.40, indicating that multicollinearity was not an issue (typical cut-offs are > 5 or 10;^49^). No effects reached significance: the main effect of State Anxiety was *p* = 0.057; the Trial × State Anxiety interaction was *p* = 0.080; and the Trial × Uncontrollable Stress interaction was *p* = 0.349. The combined model did not provide a significantly better fit to the data than either of the simpler models (see Table S.4for model comparisons). These analyses were followed by an exploratory mediation analysis that did not reach significance (see supplementary results).

### Impact of uncontrollable stress and state anxiety on proportion of probabilistic errors

We performed preregistered linear regressions examining how the nine factors predicted the proportion of probabilistic errors. The proportion of probabilistic errors was the ratio of the number of times participants made an erroneous shift away from a correctly chosen learned target after receiving probabilistic negative feedback, to the total number of times they received probabilistic negative feedback. In other words, probabilistic errors are the proportion of times participants shift away from choosing a learned target following negative feedback for a correct response, suggesting pre-emptive anticipation of a target reversal. This measure is evidently not meaningful for the signalled task. Not all participants had the opportunity to commit probabilistic errors as some never experienced probabilistic negative feedback after meeting the learning criterion of five consecutive correct trials. This reduced the sample size for this analysis to 393. The 34 excluded participants were less accurate overall (mean proportion correct = 0.329, compared to 0.535 for the included participants), and so the sample included in the probabilistic error analyses is skewed towards more accurate participants.

Uncontrollable Stress, negatively-framed State Anxiety, and positively-framed State Anxiety significantly positively predicted a greater proportion of probabilistic errors (Table 2). These significant effects were not dependent on using congeneric scores and were replicated when using the respective summed score (see supplemental table S.9). However, positively-framed State Anxiety did not survive Holm correction (*p* = 0.031, threshold *p* = 0.007). In a multiple regression model with the above three factors, only negatively-framed State Anxiety significantly predicted higher proportion of probabilistic errors (*β* = 0.047, *p* = 0.022; other *p*’s > 0.439).

**Table 2:** Linear regression model results (n = 393) for the reversal task examining the effect of each factor on probabilistic error proportions (preregistered as the ratio of probabilistic errors, and calculated as the number of times participants made an erroneous shift away from a correctly chosen target after receiving probabilistic negative feedback, divided by the total number of times they received probabilistic negative feedback), indexing a tendency to interpret negative feedback as a change in reward contingencies (i.e., a target reversal).

Model	Fixed effects	Estimate	se	*t* value	*p* value	CI lower	CI higher
**Uncontroll-able stress**	Intercept	0.341	0.015	22.647	< 0.001	0.311	0.370
	**Main effect**	**0.038**	**0.014**	**2.802**	0.005**	**0.012**	**0.065**
**State anxiety** **neg-framed**	Intercept	0.341	0.015	22.801	< 0.001	0.311	0.370
	**Main effect**	**0.051**	**0.014**	**3.575**	< 0.001**	**0.023**	**0.079**
**State anxiety** **pos-framed**	Intercept	0.341	0.015	22.556	< 0.001	0.311	0.370
	**Main effect**	**0.031**	**0.014**	**2.168**	0.031*	**0.003**	**0.060**
Trait anxiety neg-framed	Intercept	0.341	0.015	22.532	< 0.001	0.311	0.370
	Main effect	0.026	0.014	1.849	0.065	− 0.002	0.054
Trait anxiety pos-framed	Intercept	0.341	0.015	22.473	< 0.001	0.311	0.370
	Main effect	0.017	0.014	1.161	0.246	− 0.011	0.045
Lack of self-efficacy	Intercept	0.341	0.015	22.448	< 0.001	0.311	0.370
	Main effect	0.013	0.014	0.922	0.357	− 0.014	0.039
Depression	Intercept	0.341	0.015	22.510	< 0.001	0.311	0.371
	Main effect	0.021	0.014	1.478	0.140	− 0.007	0.050
Likelihood of COVID-19 risk	Intercept	0.341	0.015	22.470	< 0.001	0.311	0.370
	Main effect	− 0.015	0.014	− 1.077	0.282	− 0.043	0.013
Severity of COVID-19 risk	Intercept	0.341	0.015	22.462	< 0.001	0.311	0.371
	Main effect	0.008	0.013	0.587	0.558	− 0.018	0.033

Confidence intervals are 95%. se is the standard error of the model estimate. With a Holm correction (Holm^48^) the Uncontrollable Stress, and the State Anxiety (negatively-framed) effects remained significant (for both effects, the adjusted alpha threshold rounded to 0.006). The State Anxiety (positively-framed) main effect did not survive the Holm correction (threshold .007). Asterisks * indicate a significant effect before the Holm correction; ** indicates a Holm-corrected significant effect. A Holm correction is more powerful than a Bonferroni correction, is valid under the same assumptions, and controls the family-wise error rate. To perform the Holm correction, p-values are ordered from smallest to largest. A p-value is significant when pk < α/m + 1−k, where α is the alpha level; m is the number of p-values (18 in this case); and k is the p-value ranking.

Given that the predicted significant effect of Uncontrollable Stress was non-significant when State Anxiety was included in the model, we ran a mediation on probabilistic error proportions with Uncontrollable Stress as the predictor and State Anxiety as the mediator (Table 3). State Anxiety mediated 70% of the effect of Uncontrollable Stress on probabilistic error proportions (*p* = 0.028). Thus, uncontrollable stress was associated with a propensity to shift choices in anticipation of changes to reward contingencies, an effect which depended on current anxiety.

**Table 3:** Results from the two causal mediation analyses on reward learning slopes (estimated from the GLMM with only Trial as a fixed-effect, with by-subject random-intercepts and slopes for Trial), and the two mediation analyses on probabilistic error proportions (Prob Errors), which index the proportion of times participants make errors following probabilistic negative feedback for a correct response, after they have learned the target.

Predictor	Mediator	Outcome	*n*	Effect	Estimate	*p* value	CI lower	CI upper
Uncontrol stress	State anxiety	Learning slopes	427	ACME	− 0.020	0.048*	− 0.042	0.000
				ADE	− 0.014	0.395	− 0.046	0.019
				Total effect	− 0.034	0.009*	− 0.059	− 0.009
				Proportion mediated	0.589	0.056	− 0.023	2.353
Uncontrol stress	State anxiety	Prob errors	393	ACME	0.027	0.023*	0.004	0.051
				ADE	0.011	0.530	− 0.024	0.046
				Total effect	0.039	0.005*	0.012	0.065
				Proportion mediated	0.696	0.028*	0.088	2.462
State anxiety	Transition parameter (*tr*)	Learning slopes	427	ACME	− 0.019	0.010*	− 0.034	− 0.004
				ADE	− 0.023	0.036*	− 0.045	− 0.002
				Total effect	− 0.042	0.002*	− 0.069	− 0.016
				Proportion mediated	0.445	0.013*	0.136	0.919
State anxiety	Transition parameter (*tr*)	Prob errors	393	ACME	0.018	0.012*	0.004	0.000
				ADE	0.034	0.007*	0.009	0.019
				Total effect	0.051	< 0.001*	0.023	− 0.009
				Proportion mediated	0.341	0.013*	0.093	2.402

For both variables, we tested whether the effects of uncontrollable stress were mediated by state anxiety and whether the effects of state anxiety were mediated by the tr parameter of the HMM which estimates expected beliefs about the chance of the target changing identity from trial-to-trial (see “Computational modelling”). In other words, probabilistic errors index pre-emptive anticipation of a target reversal. State Anxiety is the negatively-framed items factor. Confidence intervals are 95%. The Average Causal Mediated Effect (ACME) is the indirect effect of the predictor on the outcome, via the mediator. The Average Direct Effect (ADE) is the unmediated effect of the predictor on the outcome. The Total Effect (ADE + ACME) is the combined effect of the predictor and mediator on the outcome. Asterisks * indicate significant effects (alpha threshold of 0.05). The sample size is indicated in the table; numbers vary in the probabilistic error analyses (n = 393) as some participants did not experience ambiguous negative feedback after learning the target and so did not have the opportunity to make probabilistic errors.

### Computational modelling

To examine possible cognitive mechanisms underlying effects of Uncontrollable Stress and State Anxiety on choices, we assessed the ability of a range of models from two families to capture trial-by-trial data (see “Materials and methods”). The first family of models includes preregistered variations of classical reinforcement learning models (RLMs) in which actions are learned through reward prediction errors and the Rescorla Wagner updating rule. However, because the RLMs were rather incompetent at explaining the observed choices, we deviated from the preregistration and additionally fitted a hidden Markov model (HMM; Fig. 2, panel D). As we did not observe any significant effects of our factors on signalled task performance, we only report modelling results for the reversal task. For completeness, model comparison results for the signalled task are shown in Table S.9. The winning model for the signalled task was an RLM, yet, even there, 34% of participants were better fitted by the HMM model.

The most parsimonious account of the reversal task data was provided by an HMM (Table 4; Fig. 2, panel D) with three free parameters (Table S.10 shows summary statistics). The identity of the target is a hidden state because it cannot be observed directly, but only indirectly through reward feedback after each choice. On each trial, the HMM estimates the probability that each of the nine images is the target given the choices and the outcome of the trial and uses this to calculate the posterior distribution over the hidden state. This likelihood is calculated differently for chosen and unchosen images and is dependent on two free parameters representing: the model’s expected probability that a reward is observed when the chosen stimulus includes the target (*q*); and the model’s expected probability that a non-reward outcome is observed when the chosen stimulus does not include the target (*p*). We note that the distribution of *p* was highly skewed towards 1, which is too high given that the notional expected probability of not obtaining a reward when non-target stimuli are chosen was 0.8. By contrast, the distribution of *q* was relatively normal but underestimated the true probability of obtaining a reward after selecting a target. After updating, the hidden states are multiplied by a transition matrix characterizing the subjective probabilistic relationship between the hidden state on the current trial and that on the next trial. This transition matrix is parameterized by a third free parameter, assuming that any new target is chosen uniformly amongst the eight other images. More formally, the off-diagonal entries of this transition matrix are set to be the transition parameter (*tr*) divided by 8. The diagonal entries are defined as 1 minus that free parameter and describe the probability that the target image remains the same on the next trial.

**Table 4:** Model comparison statistics for the reversal task using the Hierarchical Bayesian Inference toolbox (Piray et al.^50^).

Model family	Model name	# Of parameters	Model frequency	Exceedance probability
RLM	Beta-alpha	2	< 0.001	0
RLM	Beta-alpha-forget	3	0.090	0
RLM	Beta-alpha-forget-confidence	6	0.382	0.001
HMM	Hidden Markov model	3	0.528	0.999

RLM refers to Reinforcement Learning Models; HMM refers to the hidden Markov model with three parameters. Model frequency indicates the ratio of participants assigned to each model. Exceedance probability is the likelihood that each model is the most likely model, considering the possibility that differences in model evidence are due to chance.

We examined possible relationships between State Anxiety and Uncontrollable Stress and the three parameters from the HMM (q, p, and tr). The positive correlation between State Anxiety and *tr* was *r* = 0.124, *p* = 0.010 (uncorrected *p* value). No other correlations were significant. These results suggest that anxiety is associated with an increased tendency to believe that the target identity will change, shifting interpretation of negative feedback towards a change in reward contingencies rather than being due to chance. This is in line with higher anxiety being associated with a higher proportion of probabilistic errors. To test this hypothesis, we ran two exploratory mediation analyses with State Anxiety as the treatment variable and *tr* as the mediator on learning slopes, and on probabilistic errors (Table 3). The transition parameter mediated 45% of the relationship between State Anxiety and learning (*n* = 427, *p* = 0.013), and 34% of the relationship between State Anxiety and probabilistic error proportions (*n* = 393, *p* = 0.013). The above findings show that anxiety is associated with impaired reward learning via a propensity to anticipate changes to reward contingencies, an effect which depends on expected beliefs about environmental volatility.

### Test–retest reliability of task performance and model parameters from HHM

For the 49 participants who completed the probabilistic reward learning tasks twice, ICC between the two sessions was low (*ICC*(48, 48) = 0.28, *p* = 0.024, *CI* = 0.0048–0.52 for the reversal task, and *ICC*(48, 48) = 0.18, *p* = 0.092, *CI* = − 0.082 to 0.42 for the signalled task), as is the case for many cognitive tasks^1,12^. Although low test–retest reliability suggests that our task may be less suited to study individual variability and may result in underestimation of effect sizes, our ability to detect significant effects is enhanced by our relatively large sample (N = 427).

We also examined test–retest reliability for the three parameters from the winning HMM. Test–retest reliability for *p* was absent: *ICC*(48, 48) =  < 0.001, *p* = 0.500, *CI* = − 0.26 to 0.27. Test–retest for *q* was better, *ICC*(48, 48) = 0.24, *p* = 0.042, *CI* = − 0.03 to 0.49. Test–retest for the *tr* was relatively high, *ICC*(48, 48) = 0.48, *p* < 0.001, *CI* = 0.21–0.67. Importantly, although still relatively low, the reliability for the *tr* parameter was the highest we observed in the current experiment, suggesting that the computational model provides a more robust measure of task performance. This is unsurprising as this parameter showed the highest recoverability (r = 0.9096, see supplemental Fig. S.3).

## Discussion

We found that self-reported uncontrollable stress and state anxiety together predicted impaired performance in a probabilistic reversal learning task. Both uncontrollable stress and anxiety were associated with a propensity to commit probabilistic errors, reflecting incomplete learning because of enhanced anticipation of target reversals. We also found that Reinforcement Learning Models failed to capture participants’ performance in the reversal learning task whereas a Hidden Markov Model (HMM) provided a satisfactory fit to participants’ choices. The *tr* parameter of the HMM reflecting participants’ beliefs that the identity of the target image shifts from trial-to-trial mediated effects of state anxiety on reward learning and probabilistic errors. These findings suggest that uncontrollable stress and anxiety are associated with an overly volatile representation of the reward structure of the environment, promoting interpretation of probabilistic negative feedback as changes in reward contingencies, ultimately impairing reward learning in ambiguous contexts.

The negative results on the signalled task were unexpected but are concordant with the importance of probabilistic errors in driving the effects on the reversal task. In both tasks, an element of first-order uncertainty arises from the probabilistic nature of the reward structure. A second-order source of uncertainty is related to the volatility of the environment and differs between tasks. Whereas both tasks had frequent changes of reward contingencies, their occurrence was known to the participants in the signalled task but occurred silently in the reversal task. This results in negative feedback being unambiguous in the signalled task but ambiguous in the reversal task. In the latter, negative feedback could be the result of first-order uncertainty present in both tasks or a sign that the target image had changed. Accordingly, humans give more weight to unexpected outcomes (increased learning rate) in contexts with non-signalled and rapidly-changing reward contingencies^30,32^. Thus, rather than simply impairing the ability of learning the value of actions from feedback, perceived uncontrollable stress and associated anxiety appear to interfere with the ability to flexibly relearn the values of actions in the more volatile and ambiguous context of the reversal task.

We used computational modelling to examine possible cognitive mechanisms that might underpin these effects. Our preregistered RLMs did not adequately capture participants’ choices in the reversal task. Although we expected that RLMs with fixed learning rates would not recapitulate participants’ choices in the reversal task^30,51,52^, we were surprised by the poor performance of the RLM that uses estimates of confidence to update learning rates^53^. This suggests that adapting the learning rate is not sufficient to capture how participants adapted to the change in reward contingencies in our task, perhaps because of the complexities of the interactions between the three images associated with each choice. An additional consideration could be narrowing the focus of attention to the relevant category to learn a new target^44^. In sharp contrast, our HMM with only three parameters provided an excellent fit to participants’ choices in the reversal task, even though the recovered parameters do not reflect the statistics of the task. This demonstrates the potential for HMMs to understand the mechanisms underlying reward learning in complex and unstable environments and suggest a computationally simple mechanism by which the human brain infers the probability of hidden states. State anxiety positively correlated with the parameter of the HMM governing the transition matrix that determines the diffusion of hidden states between images from trial-to-trial. In participants with high state anxiety, the hidden states tend to diffuse more freely among the stimuli. This suggests that anxiety promotes imprecision in the representation of the reward structure of the task with enhanced expectation that established reward contingencies are likely to shift. In the reversal task, this belief appears to increase the tendency to interpret negative feedback as a target reversal rather than due to chance. Supporting this notion, the transition parameter mediated effects of deleterious effects of state anxiety on probabilistic error proportions and reward learning.

Our findings extend previous evidence for protective effects of perceived environmental controllability, and maladaptive effects of experiencing lack of control. For example, a sense of control in stressful environments attenuates later behavioural and neural responses to aversive stimuli^54–63^. Conversely, perceived stress uncontrollability can enhance feelings of helplessness^58^, increase stress responses, and promote passive behaviour in the face of later stressors^15,16^. Our findings suggest that, similarly to how uncontrollable stress impairs acting to avoid stress, it also can impair acting to gain rewards in ambiguous contexts. Thus, perceived uncontrollable stress may affect processes that encompass learning from both positive and aversive outcomes to make optimal choices to avoid stress or gain rewards in uncertain contexts.

Trait anxiety, state anxiety, and acute stress have all been linked to deficits in adjusting learning rates to match current environmental volatility^7,24,32,60^. Moreover, failures to adaptively adjust learning rates in volatile contexts has been linked to a more general trait negative affect factor that includes both anxiety and depressive symptoms, and this deficit generalises across learning from rewarding and aversive outcomes^34^. But there are mixed findings regarding the mechanisms underlying this altered flexibility. For example, trait anxiety has been seemingly paradoxically linked to quicker behavioural adjustments in response to punishments^31^ and to less sensitivity to negative feedback^36^. Our findings suggest that state (but not trait) anxiety is specifically associated with greater anticipation of environmental volatility under conditions of second-order uncertainty, thus increasing probabilistic errors and impairing reward learning.

One limitation is that within-subject test–retest reliability for task performance was low. Low test–retest reliability does not indicate that a task is not a replicable, valid, or a robust measure of a construct, but it does make it more difficult to detect relationships between task performance and individual differences^64^. Thus, although this issue is mitigated by our relatively large sample of 427 participants, our effect sizes may be underestimated. In future studies, using multiple tasks to obtain a composite index that reflects performance of a common latent construct such as reward learning may increase test–retest reliability^65^. Another limitation is that our preregistered exploratory factor analysis did not produce reliable factor scores, and so we instead used confirmatory factor models to obtain weighted scores based on previously established scales and subscales. A promising avenue for future work is using Computational Factor Modelling to identify and validate symptom dimensions against computationally well-defined neurocognitive processes^66,66^.

To conclude, our results show that perceived uncontrollable stress and state anxiety collectively predicted worse reward learning in the reversal task involving second order uncertainty. The computational modelling suggests that state anxiety promotes a misrepresentation of the reward structure of the environment, enhancing expectations of environmental volatility. As a result, participants with higher state anxiety tend to interpret ambiguous negative feedback as a change in reward contingencies, impairing exploitation of known reward regimes in uncertain contexts.

## Materials and methods

### Participants

500 participants were recruited via the online platform Prolific (https://www.prolific.co) with the only criteria being fluent in English. Eleven participants’ data could not be recovered from Pavlovia (see below). Nine participants were excluded based on the preregistered criterion (mean response time/RT < 300 ms). 53 participants were excluded from the reversal task because of a programming error. This left a total of 427 participants (189 female) from 47 different countries, with a mean age of 30 years (range 18–74). 50 of the same participants (49 after one exclusion) completed an identical session approximately three days later to check test–retest reliability of task performance. According to Swedish law on ethical approval of research on human participants (2003: 460), this study did not require approval from the Swedish Ethics Review Authority (https://etikprovningsmyndigheten.se/) because no personal data or biological material was collected, and we did not use any physical or mental intervention.

### Experimental task and procedure

The experiment was hosted on Pavlovia (https://pavlovia.org/) and lasted on average 28 min. Participants were given information about the experimental task and questionnaires before giving informed consent by pressing a button. They read task instructions and completed three practice games of the signalled task. After the two tasks, participants were given overall points tally, and total money earned. Lastly, they completed the mood questionnaires. Participants were paid £3.00 GPB and could earn a bonus of up to £2.30 GBP based on task performance (total average £4.39).

All participants performed two versions of a probabilistic reward learning, a three-armed bandit task (Fig. 1) adapted from Leong et al.^44^. In both tasks, the goal was to maximise monetary reward. On each trial, participants chose one of three vertical columns, each comprising three images. Images in each horizontal row were from the same class (landscape; mode of transport; animal) but were randomly shuffled within row on each trial. On any given trial, one of the images (e.g., a cat) was the target. Participants had to learn by accumulating evidence from their composite choices. Participants first completed the *signalled task* in which they were informed that the target had changed at the start of each new game of 25 trials (5 games in total). Choosing the composite stimulus with the target had a reward probability of 0.75 whereas non-target composites had a reward probability of 0.25. Participants then completed the *reversal task*, which comprised one continuous game of 125 trials in which the target changed without warning every 20–30 trials. Participants were informed about the existence of the reversals. As the reversal task was more difficult than the signalled task, we increased reward probability upon choosing the target to 0.8 (0.2 for non-targets). Participants were informed about reward probabilities for both tasks.

### Questionnaires

All questionnaires are well-validated and established measures of their respective constructs except the new Perceived Risk of COVID-19 scale that included 10 items assessing perceived potential impact of COVID-19 on oneself and others^43^. The PHQ-9 included 9 items assessing depressive symptoms rated on a 4-point Likert scale from “never” to “almost every day”^40^. The PSS included 10 items assessing perceived ability to cope with stress rated on a 5-point Likert scale from “never” to “very often”^39^. The State-Trait Anxiety Inventory (STAI) included 40 items rated on a 4-point Likert scale from “not at all” to “very much so” assessing how they felt right at that moment, and how they feel generally^41,42^. Positively worded items (e.g., “I feel comfortable”) were reverse-coded so that higher scores indicated greater stress, state and trait anxiety, depression, and perceived risk of COVID-19. We did not collect data on the use of psychiatric medications or previous diagnosis.

### Data analyses

Analyses follow the preregistration plan (https://osf.io/h8a2v) unless otherwise noted (see supplementary materials for a summary of all deviations). Code and data to reproduce all analyses is included on the OSF project page (https://osf.io/ps38n/). Our key dependent variable (DV) was accuracy. Choosing the composite stimulus that included the target image was coded as a correct response. Non-responses (fewer than 1% of trials) were recorded as errors.

Another preregistered DV was the proportion of probabilistic errors: the ratio of the number of times participants made an erroneous shift away from a correctly chosen learned target after receiving probabilistic negative feedback, to the total number of times they received probabilistic negative feedback. As preregistered, the criterion of learning was five consecutive correct trials. 34 participants never experienced probabilistic negative feedback after meeting the learning criterion and were excluded from the probabilistic error analyses. Excluded participants were less accurate (mean proportion correct = 0.329, compared to 0.535 for the included participants), and so the sample included (N = 393) is skewed towards more accurate participants.

Test–retest reliability for factor scores, mean task performance, and the estimated parameters from the winning HMM were indexed by intraclass correlation coefficient (ICC) using two-way random-effects models^45,46^.

In the preregistration we predicted that Uncontrollable Stress would be associated with fewer win-stay trials in the reversal task. We also expected perceived uncontrollable stress to result in greater sensitivity to negative feedback, which would be observed in fewer perseveration errors. Win-stay trials are choosing the target directly after being rewarded for choosing the target. Perseverative errors index the tendency to stick with choosing the previously learned target after a target reversal has occurred, despite receiving negative feedback for choosing the previous target. These dependent variables are typically used in two-armed bandit tasks with non-composite stimuli, when it is clear what stimulus participants are basing their value estimation and choice on. However, in our three-armed bandit task, a choice could be based on a prediction that any one of the three images comprising the chosen stimulus were the target. Because the images shuffle on each trial, it therefore makes less sense to examine these dependent variables with our task, and we did not analyse these DVs.

### Calculating factor scores from the questionnaire data

As preregistered, we initially used Exploratory Factor Analysis (EFA) to derive latent factor scores within and across questionnaires (see supplementary methods). However, these EFA scores were not reliable as assessed on the 49 participants who completed the scales twice 3 day apart (ICC^45,46^ ranged between 0.14 and 0.61; see Table S.1). This was surprising as the sum scores for each scale and subscale showed good test–retest reliability (ICC range: 0.75–0.95).

As the use of sum scores is not recommended, we deviated from the preregistration and calculated congeneric factor scores, using confirmatory factor analysis to produce weighted scores based on previously established structure of the questionnaires. In a congeneric model, items’ contribution to the score depends on how related the item is to the construct. Each item is allowed unique error variance and is constrained to have a variance equal to 1 and the intercept to 0. For all scales (see Table S.3), congeneric models were a better fit to the data than parallel models (equivalent to sum scores with equal contribution for all items), indicating that the weighted congeneric scores were preferred over sum scores to be used in subsequent analyses. We also determined whether each scale should be subset into previously established subscales (see supplemental methods for details). Congeneric models were fitted using the “lavaan” package in R^67^. Model comparison was done using the “nonnest2” package in R^68^.

### Generalised logistic mixed-models

To examine the effect of each Factor on learning we performed generalised logistic mixed-models (GLMMs, using the lmer R package^69^) with accuracy (0, 1) as the dependent variable, Trial (centred around 0) as a fixed-effect, including subject random intercepts and slopes for Trial. Although the preregistration stated *linear* mixed-models, a *logistic* mixed-model is appropriate for binary variables, such as accuracy (0, 1). Furthermore, we deviated from the preregistered inclusion of game as a fixed factor as we did not expect performance to linearly increase in the reversal task and it was unclear how to code this factor.

Each Factor was included as an interaction with Trial in separate models. If a significant Trial × Factor interaction was present, we compared the fit to a model without that interaction, using a chi-squared ANOVA test. If including the Trial × Factor interaction significantly improved model fit, we added, and compared, the Trial × Factor interaction term as by-subject random intercept and slope. Factors that interacted significantly with Trial were entered together into one final combined model. We checked for multicollinearity between Factor scores by calculating Variance Inflation Factors (VIFs) using the “car” package in R^70^.

We used the “mediation” package in R^71^ to perform exploratory causal mediation analyses^72^. We calculated 95% confidence intervals using 10,000 bootstrapped samples.

### Computational modelling

We fitted a range of models from two main families to the observed choices in the reversal task. As indicated in the preregistration, we fitted a range of reinforcement learning models (RLMs) to examine the cognitive mechanisms by which perceived uncontrollable stress or other latent factors might impact learning in the signalled or reversal tasks. Because the RLMs did a poor job at explaining the observed choices in the reversal task, we deviated from the preregistration and fitted two hidden Markov models (HMMs). Choices in the signalled task were not analysed with computational modelling because we did not observe any significant effects for any of the nine factor scores on performance.

### Reinforcement learning models (RLMs)

Our RLMs assume that participants learn to associate each image with a value (feature learning), based on reward feedback, and linearly combine these values to determine the value of each choice on a given trial and assumed an average value across all three stimuli (e.g., Leong et al.^44^):

1 $$V_{t} \left( {S_{i} } \right) = \mathop \sum \limits_{d} \frac{1}{3}v_{t} \left( {d,S_{i} } \right)$$

where $V_{t} \left( {S_{i} } \right)$ is the value of a composite stimulus *i* on trial *t*, and $v_{t} \left( {d,S_{i} } \right)$ is the value of image *d* on stimulus $S_{i} .$ For the signalled task, all $v$ were initialised to 0 at the beginning of each game. For the reversal task, all $v$ were initialised to 0 at the beginning of the first game. On each trial, the prediction error is calculated as the difference between the reward obtained $r_{t} \in \left\{ {0,1} \right\}$ and the value of the chosen composite stimulus, $S_{c}$, on that trial $V_{t} \left( {S_{c} } \right)$:

2 $$\delta_{t} = r_{t} - V_{t} \left( {S_{c} } \right){ }$$

We used this prediction error to update the value of the images included in the chosen composite stimulus:

3 $$v_{t + 1} \left( {d,S_{c} } \right) = v_{t} \left( {d,S_{c} } \right) + \alpha \delta_{t}$$

where $\alpha$ ($0 < \alpha < 1$) is the learning rate determining how much the future values reflect the latest experienced outcome.

Finally, we calculated the choice probability using the softmax rule:

4 $$p\left( c \right) = \frac{{e^{{\beta V_{t} \left( {S_{c} } \right)}} }}{{\mathop \sum \nolimits_{a} e^{{\beta V_{t} \left( {S_{a} } \right)}} }}$$

whereby $p\left( c \right)$ is the probability of choosing the composite stimulus *c*, *a* enumerates over all available composite stimuli, and $\beta$ ($\beta > 0$) is the inverse temperature parameter of the softmax rule determining how much choices are determined by the differences in values among stimuli.

The simplest RLM has two free parameters—learning rate ($\alpha )$, and softmax inverse temperature ($\beta$, which captures reward sensitivity at one end of the spectrum, and stochasticity in responding at the other). To improve the performance of the RLM, we deviated from the preregistration and augmented the base model with a forget parameter $\varphi$ ($0 < \varphi < 1)$ by which the value of the unselected images relaxed towards 0, the initial value (e.g., de Boer et al.^73^):

5 $$v_{t + 1} \left( {d,S_{a} } \right) = v_{t} \left( {d,S_{a} } \right) + \varphi \left( {0 - v_{t} \left( {d,S_{a} } \right)} \right) \chi_{a \ne c}$$

where the last term restricts forgetting to the non-chosen images. As indicated in the preregistration, this model was augmented to include two separate learning rates: $\alpha_{p}$ for positive $( \delta > 0$), and $\alpha_{n}$ for negative reward prediction errors ($\delta \le$ 0). Finally, the model with forget rate and two separate learning rates was augmented to include a meta-learning level (Vinckier et al.^53^). The meta-learning model computes a trial-by-trial measure of confidence $Conf_{t}$ in choice based on the absolute value of the prediction error on a given trial so that when prediction errors are smaller, confidence is higher:

6 $$Conf_{t + 1} = Conf_{t} + \gamma \left( {\left( {2 - \left| {\delta_{t} } \right|} \right)/2 - Conf_{t} } \right)$$

where $\gamma$ is the confidence learning rate, and is a free parameter. Confidence then modulates the learning rate on a trial-by-trial basis:

$$\alpha_{t} = \left( {\alpha_{p} + \kappa Conf_{t} } \right)/\left( {1 + \kappa Conf_{t} } \right)\;\;{\text{if}}\;\;\delta > 0$$

7 $$\alpha_{t} = \alpha_{n} /\left( {1 + \kappa Conf_{t} } \right)\;\;{\text{otherwise}}$$

where $\kappa$ is a free parameter determining the extent to which confidence modulates learning rate differently depending on whether the outcome received on that trial was better or worse than expected: the learning rate increases proportional to the confidence for better than expected outcomes and decreases proportionally to the confidence in worse than expected outcomes. In the preregistration, we planned to include a model with confidence modulating the softmax inverse temperature parameter. However, given the poor performance of the RLM in our task, we did not continue exploring the model space of RLMs.

Our preregistered predictions from the RLMs were:

1. For the signalled task we predicted that Uncontrollable Stress would decrease the learning rate for positive reward prediction errors (better than expected outcomes); while not affecting reward sensitivity (*β*); nor affecting learning rate for negative prediction errors (${\alpha }_{n},$ worse than expected outcomes). Alternatively, we predicted that Uncontrollable Stress may even increase the learning rate for negative prediction errors.
2. For the reversal task, in our RLMs we expected Uncontrollable Stress to increase sensitivity to negative feedback, reflected by an increased learning rate for negative prediction errors ($\alpha_{n} )$. In line with the signalled task, we predicted that Uncontrollable Stress would decrease the learning rate for positive prediction errors $(\alpha_{p}$): while not affecting reward sensitivity (*β*).
3. Moreover, we expected that Uncontrollable Stress would influence a second hierarchical level to our RLM: confidence $\left( {Conf} \right)$ in current task representations. Higher confidence was expected to modulate the free parameters of the RLM by increasing exploitation (reward sensitivity, *β*); increasing learning rate for outcomes that confirm expectations and reducing learning rate to outcomes that contradict expectations. We predicted that Uncontrollable Stress may reduce the rate of learning of confidence itself, and/or the extent to which confidence modulated these free parameters (learning rate for positive prediction errors ($\alpha_{p} )$, learning rate for negative prediction errors ($\alpha_{n} )$, and reward sensitivity, *β*).

These predictions were not explored because performance in the signalled task was not modulated by Uncontrollable Stress and the reversal task was poorly fitted by the RLM models.

### Hidden Markov models (HMMs)

HMMs differ from RLMs as they do not use feature learning to determine a cached value of each image. Instead, inference based on an HMM estimates the probability that each of the individual images is the target on a given trial. The identity of the target image is referred to as the hidden state because it cannot be observed directly but only indirectly through the rewards obtained after each choice. On each trial, the model updates the probability $\alpha_{t} \left( {f_{i} } \right)$ of each hidden state (i.e., each image $f_{i}$ being the target) upon observing the outcome as follows:

8 $$\alpha_{t + 1} \left( {f_{i} } \right) \propto \alpha_{t} \left( {f_{i} } \right)*lik_{i}$$

where $lik_{i}$ is the likelihood that image $f_{i}$ is the target and is calculated differently depending on whether the image was part of the chosen composite stimulus or not. For the chosen features:

9 $$\begin{array}{*{20}c} {lik_{i} = q} & {{\text{if}}\;r_{t} = 1} \\ {lik_{i} = 1 - q} & {{\text{otherwise}}} \\ \end{array}$$

where $0 < q < 1$ is a free parameter representing the model’s expected probability that a reward is observed when the chosen stimulus involves the target. For the unchosen features:

10 $$\begin{array}{*{20}c} {lik_{i} = 1 - p} & {{\text{if}}\;r_{t} = 1} \\ {lik_{i} = p} & {{\text{otherwise}}} \\ \end{array}$$

where $0 < p < 1$ is a free parameter representing the model’s expected probability that a non reward outcome is observed when the chosen stimulus does not involve the target.

After updating, the hidden states are renormalized, and the vector of hidden states is multiplied by the transition matrix mapping the expected probability that the hidden state on the next trial will transit from each image to any other image. All values of the 9 × 9 matrix except for the diagonal are specified as $tr/8$, $0 < tr < 1$ being a free parameter representing the model’s expected probability that the hidden state will change to any other feature from one trial to the next trial. The diagonal of the transition matrix is specified as $1 - tr.$ The diagonal thus represents the model's expected probability that the hidden state will not change from one trial to the next.

Finally, we calculated the likelihood of the choices based directly on the hidden states assuming probability matching (Herrnstein, 1997; Myers, 2014; Vulkan, 2000):

11 $$p\left( c \right) = \frac{{\mathop \sum \nolimits_{c} \alpha_{t} \left( {f_{c} } \right)}}{{\mathop \sum \nolimits_{i = 1}^{9} \alpha_{t} \left( {f_{i} } \right)}}$$

whereby $p\left( c \right)$ is the probability of choosing a composite stimulus including three chosen features *c*.

This HMM including 3 free parameters ($p$, $q$, and *tr*; see Fig. S.3 for recoverability checks) was originally augmented to include another free parameter $power$ ($0 < power <$ 5) that multiplies all hidden states $\alpha_{t} \left( {f_{i} } \right)$ before the likelihood of the choices was calculated. Thus, this parameter magnifies the differences in the hidden states and is akin to the inverse temperature of the softmax rule for RLMs and allows for under- and over-matching. However, recoverability checks (see Fig. S.4) showed that this extra parameter of the augmented model was not recoverable, so this model was not considered further.

### Model estimation and model comparison

Model parameters for both RLM and HMM were fitted and compared using the HBI toolbox (Piray et al.^50^) on MATLAB (2020b). The HBI toolbox simultaneously achieves parameter estimation and random effect’s model comparison using a variational approach (Piray et al.^50^). The HBI toolbox implements a hierarchical Bayesian approach that estimates the population distribution over the model parameters as well as the parameters of each individual subject given the population distribution, which constrains and regularizes individual subject's parameters estimates (Piray et al.^50^). The toolbox allows the best fitting model to vary across individual subjects and model comparison is done by counting the frequency of individual subjects that are best fit by each model and deriving the exceedance probability for each model (Piray et al.^50^). Moreover, by achieving concurrent parameters estimation and model comparison, the contribution of each subject to the group level estimates of the parameters are weighted by the degree to which a given model is likely to be the underlying model for that subject (Piray et al.^50^). See Table S.11 for the results of the sequential model comparison.

Our preregistration specified we would use RStan, however HBI allowed easier implementation of the HMMs, so we used HBI for all models. Both methods are hierarchical Bayesian approaches but whereas the HBI uses a variational Bayes approach to estimate the posterior probabilities of the parameter, the RStan uses Monte Carlo Markov Chain (MCMC) sampling to obtain the full distribution. Similarly, we deviated from the preregistration by not testing models in which individual-level parameters drawn from the group-level normal distributions were allowed to vary according to the subject score on perceived control (as suggested in Moutoussis et al.^28^). Instead, we performed correlations between the estimated parameters and the factor scores outside of the models.

### Recoverability analysis

To ascertain that we were able to recover the different models that we tested, we simulated five data sets for the reversal task. For these simulations, we used one of the following generative models: (1) base RLM model, (2) base RLM model with forget parameters, (3) base RLM model with forget parameter and confidence modulation of learning rate, (4) base HMM, and (5) base HMM with power parameter. For each generative model, we sampled 1000 combinations of the parameters using the mean and variance estimated at the group level for that model. As HBI weights the contribution of each subject to the group-level estimates of the parameters by the degree to which a given model is likely to be the underlying model for that subject, we did not have reliable parameter estimates for models that were very unlikely. To obtain parameters for those models, we estimated them by themselves, not being compared to any other model.

For each simulated data set, we then fitted all five models and performed model comparison using the HBI toolbox. We then constructed a confusion matrix (see Fig. S.5) and performed correlations of the generative parameters against the recovered parameters (see Figs. S.3 and S.4 for HMM3 and HMM4). We also checked the correlation between the generative parameters and the recovered parameters (see Figs. S3 and S4).

### Supplementary Information

Supplementary Information.

## Data Availability

Full data and code to run the task and reproduce all analyses are included on OSF (https://osf.io/ps38n/).
