# Supplementary material for: Anxiety associated with perceived uncontrollable stress enhances expectations of environmental volatility and impairs reward learning
Source: Sci Rep. 2023 Oct 27;13:18451. doi: 10.1038/s41598-023-45179-z (PMC10611750; doi:10.1038/s41598-023-45179-z)

Anxiety associated with perceived uncontrollable stress enhances expectations of environmental volatility and impairs reward learning.

Marc Guitart-Masip, Amy Walsh, Peter Dayan, Andreas Olsson

Supplementary Information

Summary of the deviations from the preregistration

Here we list the deviations from the preregistration. Details on the motivation for each change is given in the main text or the supplementary methods.

- Instead of using Exploratory Factor Analysis to derive factors from the questionnaire data, we used congeneric factor analysis, a confirmatory factor model, based on previously established subscales to obtain reliable weighted factor scores.
- Two preregistered dependent variables were not considered: number of win stay trials, and perseveration errors.
- The preregistration stated *linear* mixed-models but we used *logistic* mixed-model to study the effects of the factors on accuracy.
- The preregistration stated that we would include game as a fixed-effect for the GLMM analysis of the reversal task, but this factor was not included.
- We used exploratory mediation analysis to study the relationship between significant factors in the GLMM.
- We deviated from the preregistration and augmented the base Reinforcement Learning Model (RLM) with a forget parameter φ $(0 < \varphi < 1)$.
- We did not include a model with confidence modulating the softmax inverse temperature parameter.
- We did not test the hypotheses that we preregistered for the RLM models and instead used an exploratory Hidden Markov Model to characterise the effects of the factors on observed choices.
- Instead of using RStan, we used HBI toolbox to fit and compare the models.

- Instead of testing models in which individual-level parameters drawn from the group-level normal distributions were allowed to vary according to the subject score on perceived controllability, we tested for the correlation with the parameters outside the model.

Supplementary methods

Exploratory factor analysis

As preregistered, we initially used Exploratory Factor Analysis (EFA) to derive latent factor scores within and across questionnaires. EFA was performed using the "fa" function in the "psych" R package [67] to find latent factors present within and across these questionnaires. The Kaiser, Meyer, Olkin (KMO) measure of sampling adequacy was close to 1 ($KMO = 0.96$) suggesting that the sum of partial correlations was not large relative to the sum of correlations, meaning that the data should result in distinct and reliable factors (Kaiser, 1970). Moreover, Bartlett's test was significant ($\chi2 = 18801$, $df = 2346$, $p < .001$), indicating that the residual correlations were all zero, also suggesting that the data was appropriate for EFA. Because the data were not normally distributed (Shapiro-Wilk $mvw = 0.98$, $p < .001$) we used the ordinary least squares method to find the minimum residual solution ("minres" method) which provides results similar to the maximum likelihood method, without assuming multivariate normal distribution [3]. We used oblique ("oblimin") rotation because we expected our factors to be correlated.

There are many possible ways to determine the appropriate number of factors in an EFA (see [3,4]). As we did not preregister this detail, we tested different approaches. We first ran an EFA with eight-factors, then seven, then six, and lastly five-factors. All four solutions provided a similarly good fit to the data (e.g., [5]) with root mean square error of approximation (RSMEA) of 0.057 (five- and six-factors); 0.058 (seven-factors) and 0.061 (eight-factors). The clearest, meaningful, yet parsimonious structure was a seven-factor model, which was consistent with the parallel analysis [6] which compares the scree of factors of the observed data with a random data matrix of the same size to estimate the appropriate number of factors [3]. Factor scores were obtained using the Thurstone regression method[7,8].

However, these EFA scores were not reliable as assessed on the 49 participants who completed the scales twice 3 day apart (ICC[9,10] ranged between 0.14 and 0.61; see Table S.1). This was surprising as the sum scores for each scale and subscale showed good test-retest reliability (ICC range: 0.75 to 0.95).

Congeneric factor analysis

As the use of sum scores is not recommended, we deviated from the preregistration and calculated congeneric factor scores, using confirmatory factor analysis to produce weighted scores based on previously established structure of the questionaires[11]. In a congeneric model, items' contribution to the score depends on how related the item is to the construct. Each item is allowed unique error variance, and is constrained to have a variance equal to 1 and the intercept to 0. For all scales (see Table S.3), congeneric models were a better fit to the data than parallel models (equivalent to sum scores with equal contribution for all items), indicating that the weighted congeneric scores were preferred over sum scores to be used in subsequent analyses. We also determined whether each scale should be subset into previously established subscales.

For the Perceived Stress Scale, a two-factor model was a better fit to our data than a one-factor model, in line with previous findings[12–16]. Uncontrollable Stress (also referred to as Perceived Helplessness in the previous literature) includes six negatively-framed items regarding the impact of perceived uncontrollable stress (e.g., "In the last month, how often have you been angered because of things that were outside of your control?"). Lack of Self-efficacy includes four positively-framed items regarding perceived ability to cope with stressors (e.g., "In the last month, how often have you been able to control irritations in your life?". The two stress factors significantly correlated with one another, $r(425) = 0.636$, $p < .001$. For the Perceived Risk of COVID-19 scale, a two-factor model (Perceived *Likelihood* and Perceived *Severity* of COVID-19 Risk) was a better fit than a one-factor model. The two COVID-19 factors significantly correlated with one another, $r(425) = 0.506$, $p < .001$. Although the PHQ-9 is often considered to be a one-factor construct, a two-factor structure with a cognitive and affective factor, and a somatic factor can also be a good fit (e.g., [17]), and was a better fit to our data than a one-factor model. But because the two factors were so highly correlated, $r(425) = 0.772$, $p < .001$, for the sake of parsimony, we treated Depression as one factor.

The State and Trait Anxiety Inventory scales can be further divided into negatively-framed items indexing the presence of anxiety symptoms (e.g., "I feel upset"), and positively-framed items indexing the absence of anxiety symptoms (e.g., "I feel calm") which were reverse-coded so higher scores indicate, for example, less calm. Consistent with previous work [18,19], a four-factor model with State Anxiety (negatively-framed items), State Anxiety (positively-framed items), Trait Anxiety (negatively-framed items), and Trait Anxiety (positively-framed items) was a better fit than a two-factor Trait and State Anxiety model. The State Anxiety negatively-framed and positively-framed factors significantly correlated with one another, $r(425) = 0.645$, $p < .001$; as did the Trait Anxiety negatively-framed and positively-framed factors, $r(425) = 0.664$, $p < .001$.

Congeneric models were fitted using the "lavaan" package in R[20]. Model comparison was done using the "nonnest2" package in R[21].

References

1. Revelle, W. *psychTools:Tools to Accompany the 'psych; Package for Psychological Research*. (Northwestern University, 2021).

2. Bartlett, M. S. THE EFFECT OF STANDARDIZATION ON A χ 2 APPROXIMATION IN FACTOR ANALYSIS. *Biometrika* **38**, 337–344 (1951).

3. Schmitt, T. A. Current Methodological Considerations in Exploratory and Confirmatory Factor Analysis. *Journal of Psychoeducational Assessment* **29**, 304–321 (2011).

4. Costello, A. B. & Osborne, J. Best practices in exploratory factor analysis: four recommendations for getting the most from your analysis. doi:10.7275/JYJ1-4868.

5. Finch, W. H. Using Fit Statistic Differences to Determine the Optimal Number of Factors to Retain in an Exploratory Factor Analysis. *Educational and Psychological Measurement* **80**, 217–241 (2020).

6. Horn, J. L. A rationale and test for the number of factors in factor analysis. *Psychometrika* **30**, 179–185 (1965).

7. DiStefano, C., Zhu, M. & Mîndrilã, D. Understanding and Using Factor Scores: Considerations for the Applied Researcher. doi:10.7275/DA8T-4G52.

8. Thurstone, L. L. *The vectors of mind: Multiple-factor analysis for the isolation of primary traits.* (University of Chicago Press, 1935). doi:10.1037/10018-000.

9. McGraw, K. O. & Wong, S. P. Forming inferences about some intraclass correlation coefficients. *Psychological Methods* **1**, 30–46 (1996).

10. Shrout, P. E. & Fleiss, J. L. Intraclass correlations: Uses in assessing rater reliability. *Psychological Bulletin* **86**, 420–428 (1979).

11. McNeish, D. & Wolf, M. G. Thinking twice about sum scores. *Behav Res* **52**, 2287–2305 (2020).

12. Andreou, E. *et al.* Perceived Stress Scale: Reliability and Validity Study in Greece. *IJERPH* **8**, 3287–3298 (2011).

13. Golden-Kreutz, D. M., Browne, M. W., Frierson, G. M. & Andersen, B. L. Assessing Stress in Cancer Patients: A Second-Order Factor Analysis Model for the Perceived Stress Scale. *Assessment* **11**, 216–223 (2004).

14. Khalili, R., Sirati Nir, M., Ebadi, A., Tavallai, A. & Habibi, M. Validity and reliability of the Cohen 10-item Perceived Stress Scale in patients with chronic headache: Persian version. *Asian Journal of Psychiatry* **26**, 136–140 (2017).

15. Nielsen, M. G. *et al.* The construct validity of the Perceived Stress Scale. *Journal of Psychosomatic Research* **84**, 22–30 (2016).

16. Roberti, J. W., Harrington, L. N. & Storch, E. A. Further Psychometric Support for the 10-Item Version of the Perceived Stress Scale. *Journal of College Counseling* **9**, 135–147 (2006).

17. Boothroyd, L., Dagnan, D. & Muncer, S. PHQ-9: One factor or two? *Psychiatry Research* **271**, 532–534 (2019).

18. Spielberger, C. D. Test Anxiety Inventory. in *The Corsini Encyclopedia of Psychology* (eds. Weiner, I. B. & Craighead, W. E.) 1–1 (Wiley, 2010). doi:10.1002/9780470479216.corpsy0985.

19. Vigneau, F. & Cormier, S. The Factor Structure of the State-Trait Anxiety Inventory: An Alternative View. *Journal of Personality Assessment* **90**, 280–285 (2008).

20. Rosseel, Y. **lavaan** : An *R* Package for Structural Equation Modeling. *J. Stat. Soft.* **48**, (2012).

21. Vuong, Q. H. Likelihood Ratio Tests for Model Selection and Non-Nested Hypotheses. *Econometrica* **57**, 307 (1989).

Supplementary results

Mediation analysis

The fact that both Trial x Uncontrollable Stress and Trial x State Anxiety interactions were significant in separate models, but only the State Anxiety interaction was close to significant in the combined model suggests that variability in State Anxiety may *explain* the effect of Uncontrollable Stress on learning. To test this possibility, we implemented an exploratory (not preregistered) causal mediation analysis (Imai et al., 2010) with Uncontrollable Stress as the predictor and State Anxiety as the mediator (Table 2). To obtain a summary measure of learning we extracted random slopes (range: -0.629 to 0.943) for Trial for each participant from the simplest GLMM on accuracy with only Trial as a fixed and random-effect. Although the total effect of State Anxiety and Uncontrollable Stress together was significant (p=.009), the mediation did not reach significance (p=.056). Thus, although both factors together impact learning, we cannot make strong inferences regarding a mediation effect.

Learning speed

For both tasks, we performed preregistered multiple linear regressions examining how the nine factors predicted learning speed (number of trials to learning defined by a preregistered criterion of 5 consecutive correct trials). We expected that Uncontrollable Stress would be associated with slower learning. However, none of the factors predicted slower learning in either task (Table S.7; S.8).

Supplementary tables

Table S.1. Exploratory Factor Analysis (EFA) factor score test-retest Intraclass Correlations (ICCs) between session 1 and session 2 for scores from the seven-factor EFA (n = 49). Confidence Intervals are 95%. The ICC estimates were remarkably low, and so we opted to use confirmatory factor models based on previously established scales and subscales instead.

factor	ICC estimate	df	p	CI lower	CI upper
Anxiety pos-framed	0.518	48, 48	<0.001	0.323	0.671
State Anxiety neg-framed	0.301	48, 48	0.017	0.072	0.501
Trait Anxiety neg-framed	0.613	48, 48	<0.001	0.442	0.741
Depression	0.486	48, 48	<0.001	0.284	0.646
Uncontrollable Stress	0.556	48, 48	<0.001	0.371	0.700
Likelihood of COVID-19 Risk	0.139	48, 48	0.168	-0.099	0.362
Severity of COVID-19 Risk	0.373	48, 48	0.004	0.152	0.559

Table S.2. Model comparisons of the fit of non-nested one and two-factor parallel and congeneric models to obtain the factor scores (using the Vuong test in the "nonnest2" package in R). We used previously established scales (one-factor models) and subscales (two and four-factor models; see McNeish & Wolf, 2020) to determine contributing items. Parallel models create linear transformations of the sum scores. Congeneric models produce weighted scores based on how related each item is to the construct. LRT is the likelihood ratio test statistic (values below 0 indicate the second model is a better fit; values above 0 indicate the first model is a better fit) and the p *values test the significance of the LRT. BIC is Bayesian Information*

Criterion. CI lower and CI upper indicate 95% confidence intervals surrounding the difference between BICs for each model comparison.

scale	model type	model comparison	LRT	*p*	BIC model 1	BIC model 2	CI lower	CI upper
PSS Stress	parallel	one vs two factors	2.01	0.978	10948	11061	-217.75	-8.54
	congeneric	one vs two factors	-3.76	<0.001	10930	10835	42.11	146.89
	one-factor	parallel vs congeneric	5.44	>0.999	10948	10930	-27.81	63.65
	two-factor	parallel vs congeneric	-8.51	<0.001	11061	10835	148.52	302.61
PHQ-9	parallel	one vs two factors	9.10	>0.999	9284	9664	-460.75	-299.57
	congeneric	one vs two factors	-2.52	0.006	9204	9167	3.50	70.94
	one-factor	parallel vs congeneric	-6.40	<0.001	9284	9204	25.76	134.12
	two-factor	parallel vs congeneric	-13.83	<0.001	9664	9167	413.13	581.51
Covid Risk	parallel	one vs two factors	-2.02	0.022	17763	17650	-2.40	228.62
	congeneric	one vs two factors	-4.28	<0.001	17350	17223	65.89	187.65
	one-factor	parallel vs congeneric	-8.44	<0.001	17763	17350	291.81	534.13
	two-factor	parallel vs congeneric	-9.87	<0.001	17650	17223	320.23	533.02
	parallel	two vs four factors	-4.45	<0.001	38295	37433	476.66	1247.59

scale	model type	model comparison	LRT	*p*	BIC model 1	BIC model 2	CI lower	CI upper
STAI Anxiety	congeneric	two vs four factors	-9.08	<0.001	38056	36547	1176.21	1840.79
	two-factor	parallel vs congeneric	-10.12	<0.001	38295	38056	101.58	377.41
	four-factor	parallel vs congeneric	-14.13	<0.001	37433	36547	694.98	1076.74

Table S.3. Congeneric factor score test-retest Intraclass Correlations (ICCs) between session 1 and session 2. Scores were created from the confirmatory congeneric latent factor models based on previously established scales and subscales (n = 49). Confidence Intervals are 95%.

subscale	questionnaire	ICC estimate	df	*p*	CI lower	CI upper
State Anxiety neg-framed	STAI State	0.747	48, 48	<0.001	0.622	0.835
State Anxiety pos-framed		0.812	48, 48	<0.001	0.713	0.879
Trait Anxiety neg-framed	STAI Trait	0.883	48, 48	<0.001	0.786	0.932
Trait Anxiety pos-framed		0.867	48, 48	<0.001	0.782	0.918
Depression	PHQ-9	0.932	48, 48	<0.001	0.893	0.957
Uncontrollable Stress	Perceived Stress Scale	0.872	48, 48	<0.001	0.802	0.919
Lack of Self-efficacy		0.836	48, 48	<0.001	0.740	0.897
Likelihood of COVID-19 Risk	Covid Risk	0.835	48, 48	<0.001	0.747	0.894
Severity of COVID-19 Risk		0.791	48, 48	<0.001	0.683	0.865

Table S.4. Results of the chi-squared ANOVA model comparisons comparing each of the nine single factor Generalised Logistic Mixed Models (GLMMs) for the reversal task to the null model (with only Trial as a fixed-effect, and by-subject random intercepts and slopes for Trial); and comparing the combined model with State Anxiety (negatively-framed) x Trial and Uncontrollable Stress x Trial interactions to the two significant single factor (State Anxiety (negatively-framed) x Trial and Uncontrollable Stress x Trial) models; and comparing the two significant single factor (State Anxiety (negatively-framed) x Trial and Uncontrollable Stress x Trial) models to the same models with the addition of the interaction terms as by-subject random intercepts and slopes. AIC = Akaike information criterion; BIC = Bayesian information criterion; logLik = log likelihood; Chisq = chi-square statistic. Asterisks indicate significant effects ($p < .050$*).*

model comparison	*df*	AIC	BIC	logLik	Chisq	*p* value
null		71624	71669	-35807		
null vs Self-efficacy	2	71628	71690	-35807	0.343	0.842
null vs Depression	2	71627	71689	-35806	1.689	0.430
null vs Likely COVID-19 Risk	2	71627	71689	-35806	1.491	0.475
null vs Severity COVID-19 Risk	2	71628	71690	-35807	0.587	0.746
null vs Trait Anxiety neg-framed	2	71625	71687	-35806	3.306	0.191
null vs State Anxiety pos-framed	2	71624	71686	-35805	4.300	0.116
null vs Trait Anxiety pos-framed	2	71627	71690	-35807	1.110	0.574
null vs State Anxiety neg-framed	2	71618	71680	-35802	10.590	0.005*
State Anxiety neg-framed vs random-effects model	7	71630	71755	-35801	1.723	0.974
null vs Uncontrollable Stress	2	71621	71684	-35804	7.237	0.027*
Uncontrollable Stress vs random-effects model	7	71632	71757	-35802	3.097	0.876

model comparison	*df*	AIC	BIC	logLik	Chisq	*p* value
Combined model vs State Anxiety	2	71621	71701	-35801	0.960	0.619
Combined model vs Uncontrollable Stress	2	71621	71701	-35801	4.314	0.116

Table S.5. Results of the reversal task Generalised Logistic Mixed Model (GLMM) with Uncontrollable Stress x Trial and State Anxiety x Trial interaction terms, with by-subject random intercepts and slopes for Trial. Confidence intervals are 95%. Log odds estimates can be transformed into odds ratios by exponentiating the value. The factor State Anxiety includes the negatively-framed items from the STAI-State questionnaire only. The factor Uncontrollable Stress includes the items from the subscale of the Perceived Stress Scale (also referred to in previous literature as Perceived Helplessness).

fixed-effects	log odds estimate	CI lower	CI upper	*p* value
intercept	0.102	0.053	0.152	<0.001
Trial	0.357	0.321	0.393	<0.001
Uncontrollable Stress	-0.008	-0.067	0.052	0.798
Anxiety	-0.060	-0.122	0.002	0.057
Uncontrollable Stress x Trial	-0.021	-0.064	0.023	0.349
Anxiety x Trial	-0.040	-0.085	0.005	0.080

Table S.6. Generalised Logistic Mixed Model (GLMM) results for the signalled task: The table shows results from the nine separate models with Factor Score x Trial interactions. Confidence

intervals are 95%. se is the standard error of the log odds estimate. Significant fixed-effects and interactions are shown in bold. Log odds estimates can be transformed into odds ratios by exponentiating the value. p *values are uncorrected for multiple comparisons. No effects were significant.*

model	fixed effects	estimate	se	*z* value	*p* value	CI lower	CI higher
State Anxiety neg-framed	main effect	-0.040	0.029	-1.395	0.163	-0.096	0.016
	State Anxiety neg-framed x Trial	-0.023	0.014	-1.652	0.099	-0.050	0.004
Uncontrollable Stress	main effect	-0.028	0.027	-1.035	0.301	-0.082	0.025
	Uncontrollable Stress x Trial	-0.008	0.013	-0.591	0.554	-0.034	0.018
Lack of Self-efficacy	main effect	0.009	0.027	0.354	0.723	-0.043	0.062
	Lack of Self-efficacy x Trial	0.004	0.013	0.299	0.765	-0.021	0.029
Depression	main effect	0.003	0.028	0.102	0.919	-0.053	0.059
	Depression x Trial	-0.006	0.014	-0.471	0.638	-0.033	0.020
Likely COVID-19 Risk	main effect	-0.004	0.028	-0.132	0.895	-0.059	0.052

model	fixed effects	estimate	se	*z* value	*p* value	CI lower	CI higher
	Likely COVID-19 Risk x Trial	-0.013	0.014	-0.929	0.353	-0.039	0.014
Severity COVID-19 Risk	main effect	-0.027	0.025	-1.065	0.287	-0.076	0.023
	Severity COVID-19 Risk x Trial	-0.012	0.012	-0.965	0.335	-0.036	0.012
Trait Anxiety neg-framed	main effect	-0.019	0.028	-0.685	0.493	-0.075	0.036
	Trait Anxiety neg-framed x Trial	-0.011	0.014	-0.785	0.433	-0.038	0.016
State Anxiety pos-framed	main effect	-0.028	0.029	-0.961	0.337	-0.084	0.029
	State Anxiety pos-framed x Trial	-0.004	0.014	-0.258	0.796	-0.031	0.024
Trait Anxiety pos-framed	main effect	-0.019	0.029	-0.671	0.502	-0.075	0.037
	Trait Anxiety pos-framed x Trial	0.007	0.014	0.503	0.615	-0.020	0.034

Table S.7. Linear regression model results for the reversal task examining the effect of each factor on number of trials to learning (preregistered as five correct trials in a row), indexing learning speed. No factors significantly predicted learning speed. Confidence intervals are 95%. se is the standard error of the model estimate.

model	fixed effects	estimate	se	*t* value	*p* value	CI lower	CI higher
Uncontroll-able Stress	intercept	16.240	0.228	71.342	<0.001	15.794	16.686
	main effect	0.308	0.210	1.466	0.143	-0.104	0.720
State Anxiety neg-framed	intercept	16.240	0.228	71.380	<0.001	15.794	16.686
	main effect	0.353	0.219	1.612	0.108	-0.076	0.781
State Anxiety pos-framed	intercept	16.240	0.228	71.267	<0.001	15.794	16.687
	main effect	0.246	0.220	1.118	0.264	-0.186	0.679
Trait Anxiety neg-framed	intercept	16.240	0.228	71.323	<0.001	15.794	16.687
	main effect	0.301	0.217	1.384	0.167	-0.125	0.727
Trait Anxiety pos-framed	intercept	16.240	0.228	71.210	<0.001	15.793	16.687
	main effect	0.164	0.218	0.753	0.452	-0.264	0.592
Lack of Self-efficacy	intercept	16.240	0.228	71.162	<0.001	15.793	16.688
	main effect	0.003	0.204	0.015	0.988	-0.398	0.404
Depression	intercept	16.240	0.228	71.213	<0.001	15.793	16.687
	main effect	0.169	0.218	0.776	0.438	-0.258	0.596

model	fixed effects	estimate	se	*t* value	*p* value	CI lower	CI higher
Likelihood of COVID-19 Risk	intercept	16.240	0.228	71.250	<0.001	15.794	16.687
	main effect	0.221	0.216	1.024	0.306	-0.202	0.645
Severity of COVID-19 Risk	intercept	16.240	0.228	71.165	<0.001	15.793	16.688
	main effect	0.035	0.193	0.183	0.855	-0.343	0.414

*Table S.8. Robustness check on the Generalised Logistic Mixed Model (GLMM) results for the reversal task using summed scores instead of congeneric factor scores: Results from the two separate models that are significant in table 1 with Summed Scores x Trial interactions. Confidence intervals are 95%. se is the standard error of the log odds estimate. * Indicates a significant effect.*

model	*fixed effects*	*estimate*	*se*	*z value*	*p value*	*CI lower*	*CI higher*
State Anxiety neg-framed	*main effect*	*-0.010*	*0.004*	*-2.712*	*0.007**	*-0.017*	*-0.003*
	State Anxiety neg-framed x Trial	*-0.008*	*0.003*	*-2.992*	*0.003**	*-0.013*	*-0.003*
Stress Uncontrol	*main effect*	*-0.010*	*0.005*	*-1.946*	*0.052*	*-0.021*	*0.000*
	Stress Uncontrol x Trial	*-0.011*	*0.004*	*-2.749*	*0.006**	*-0.018*	*-0.003*

*Table S.9. Robustness check on the linear regression analysis on probabilistic error proportion using summed scores instead of congeneric factor scores: Results from the three models that are significant in table 3. Confidence intervals are 95%. se is the standard error of the model estimate. * indicates a significant effect.*

model	*fixed effects*	*estimate*	*se*	*t value*	*p value*	*CI lower*	*CI higher*
Stress Uncontrol	*intercept*	*0.231*	*0.041*	*5.699*	*<0.001*	*0.152*	*0.311*
	main effect	*0.009*	*0.003*	*2.902*	*0.004**	*0.003*	*0.015*
State Anxiety neg-framed	*intercept*	*0.197*	*0.044*	*4.535*	*<0.001*	*0.112*	*0.283*
	main effect	*0.007*	*0.002*	*3.517*	*<0.001**	*0.003*	*0.011*
State Anxiety pos-framed	*intercept*	*0.238*	*0.049*	*4.814*	*<0.001*	*0.141*	*0.335*
	main effect	*0.004*	*0.002*	*2.184*	*0.030**	*0.000*	*0.008*

Table S.10. Linear regression model results for the ***signalled*** *task examining the effect of each factor on number of trials to learning (preregistered as five correct trials in a row), indexing learning speed. No factors significantly predicted learning speed. Confidence intervals are 95%. se is the standard error of the model estimate.*

model	fixed effects	estimate	se	*t* value	*p* value	CI lower	CI higher
Uncontroll-able Stress	intercept	16.429	0.227	72.425	<0.001	15.984	16.873
	main effect	0.228	0.210	1.088	0.277	-0.183	0.639
State Anxiety neg-framed	intercept	16.429	0.227	72.482	<0.001	15.984	16.873
	main effect	0.297	0.218	1.361	0.174	-0.131	0.724
State Anxiety pos-framed	intercept	16.429	0.227	72.473	<0.001	15.984	16.873
	main effect	0.290	0.219	1.324	0.186	-0.139	0.720
Trait Anxiety neg-framed	intercept	16.429	0.227	72.333	<0.001	15.983	16.874
	main effect	0.070	0.217	0.322	0.748	-0.355	0.495
Trait Anxiety pos-framed	intercept	16.429	0.227	72.401	<0.001	15.984	16.873
	main effect	0.206	0.217	0.947	0.344	-0.220	0.632
Lack of Self-efficacy	intercept	16.429	0.227	72.328	<0.001	15.983	16.874
	main effect	0.044	0.204	0.218	0.828	-0.355	0.443
Depression	intercept	16.429	0.227	72.325	<0.001	15.983	16.874
	main effect	0.019	0.217	0.086	0.932	-0.407	0.444

model	fixed effects	estimate	se	*t* value	*p* value	CI lower	CI higher
Likelihood of COVID-19 Risk	intercept	16.429	0.227	72.329	<0.001	15.983	16.874
	main effect	-0.051	0.215	-0.235	0.814	-0.473	0.371
Severity of COVID-19 Risk	intercept	16.429	0.227	72.337	<0.001	15.983	16.874
	main effect	0.074	0.192	0.382	0.703	-0.304	0.451

Table S.11. Model comparison for the ***signalled*** *task using the Hierarchical Bayesian Inference toolbox (Piray et al., 2019). RLM refers to Reinforcement Learning Models; HMM refers to the hidden Markov model with three parameters. Model frequency indicates the ratio of participants assigned to each model. Exceedance probability is the likelihood that each model is the most likely model, considering the possibility that differences in model evidence are due to chance.*

model family	model name	# of parameters	model frequency	exceedance probability
RLM	beta-alpha	2	0	0
RLM	beta-alpha-forget	3	0.622	1
RLM	beta-alpha-forget-conf	6	0	0
HMM	hidden Markov model	3	0.378	0

Table S.12. Summary statistics (25th, 50th and 75th percentiles) for the parameters from the winning hidden Markov model with three parameters, for the reversal task. The parameter p *represents an estimate of the expected probability that a non-reward outcome is observed when the chosen stimulus does not involve the target (the actual value being 0.8);* q *represents an estimate of the expected probability that a reward outcome is observed when the chosen stimulus does include the target (the actual value being 0.8); and the parameter* tr *represents the expectation that the identity of the target will shift to another image on the next trial.*

percentile	*p*	*tr*	*q*
25%	0.993	0.081	0.349
50%	0.996	0.127	0.528
75%	0.997	0.204	0.689

Table S.13. Each sequential computational model comparison for the reversal task, including model frequency and exceedance probability estimates. Models: ba = beta-alpha; baf = beta-alpha-forget; bafc = beta-alpha-forget-conf; HMM = hidden Markov model with 3 free parameters. Model frequency indicates the ratio of participants assigned to each model. Exceedance probability is the likelihood that each model is the most likely model, considering the possibility that differences in model evidence are due to chance.

statistic	model comparison	beta-alpha	beta-alpha-forget	beta-alpha-forget-conf	hidden Markov model
model frequency	ba vs baf	0.052	0.948		
	ba vs baf vs bafc	0.043	0.353	0.604	
	bafc vs HMM			0.414	0.586
exceedance probability	ba vs baf	< 0.001	1		
	ba vs baf vs bafc	0	0	1	
	bafc vs HMM			< 0.001	1

Supplementary figures

Figure S.1. Distribution of performance measures: learning slopes extracted from the null GLMM (z scored by default), the number of games learned, average trial to learning criterion for participants learning at least one game (N=398), and proportion of probabilistic errors for participants that experienced negative feedback at least once after reaching learning criterion (N=393).

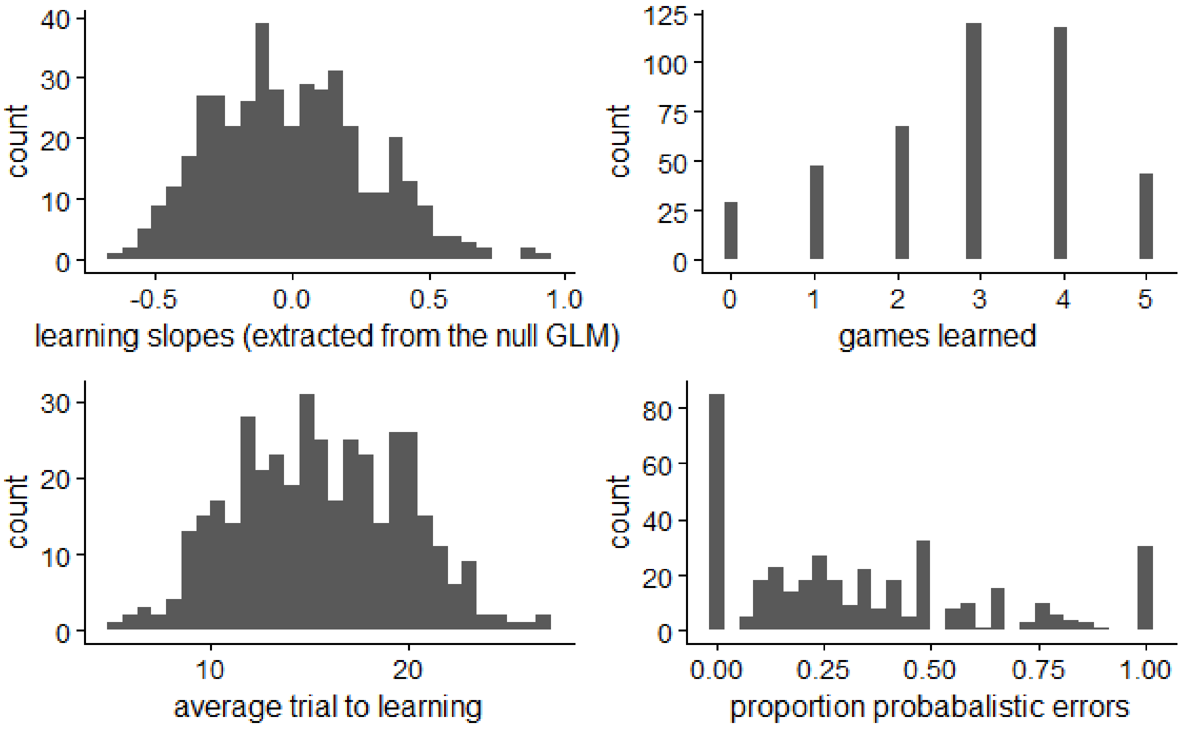

Figure S.2. Distribution of sum scores for the state and trait anxiety as well as the perceived stress scale. The black lines indicate the mean of our sample for each scale. For the state and trait anxiety, the red line displays the cutoff that is used to indicate clinically significant symptoms of anxiety [6]. We note that many of our participants scored above this threshold, which probably reflects increased levels of anxiety due to the covid pandemic that had just started when the data was collected. For the perceived stress scale, the yellow line indicate the separation between low and moderate stress, and the cyan line indicates the separation between moderate and high stress[7].

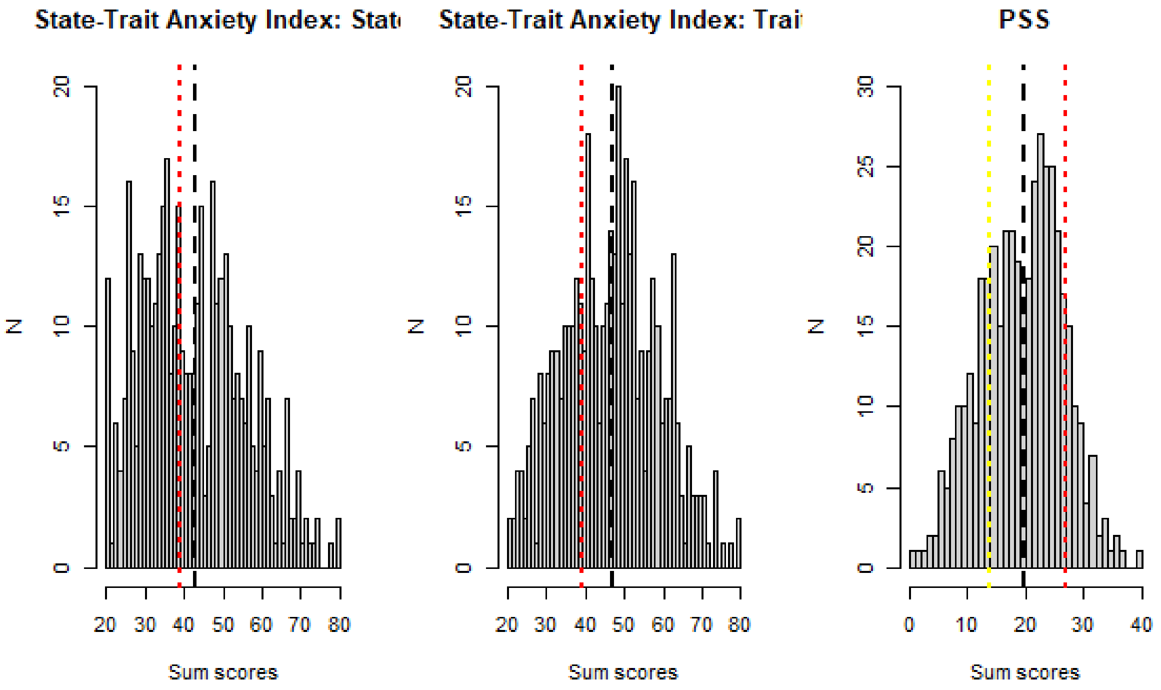

Figure S.3. Correlations between observed and generative parameter estimates for each of the three parameters of the winning hidden Markov model (pb r=0.6975; trans r=0.9096; qg r=0.8554)

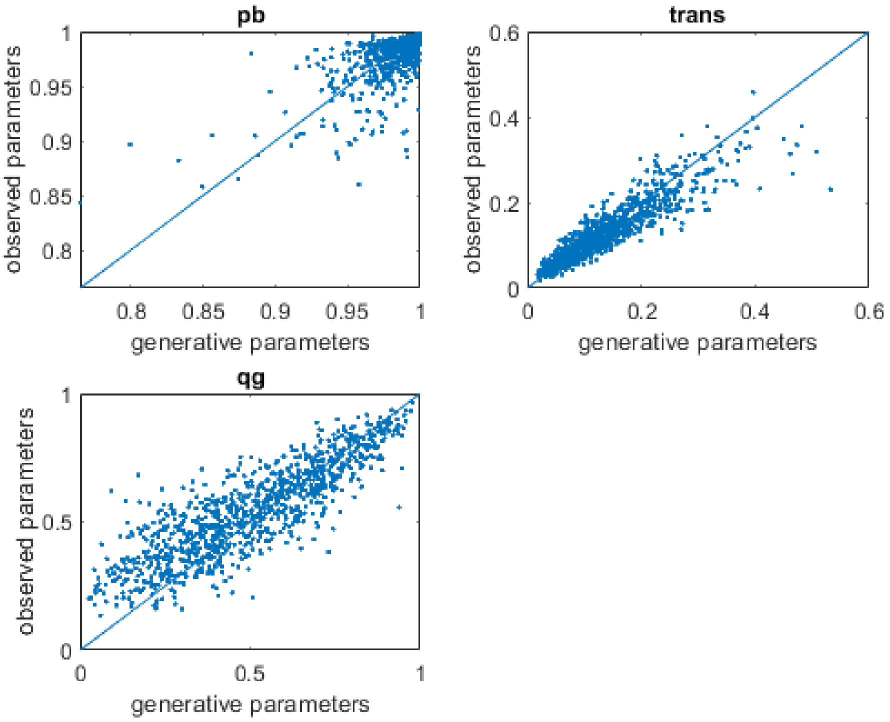

Figure S.4. Correlations between observed and generative parameter estimates for the hidden Markov model with four parameters (pb r=0.5271; trans r=0.8237; qg=0.8114; pow r=0.1578). The power parameter was not recoverable, and so this model was not considered further.

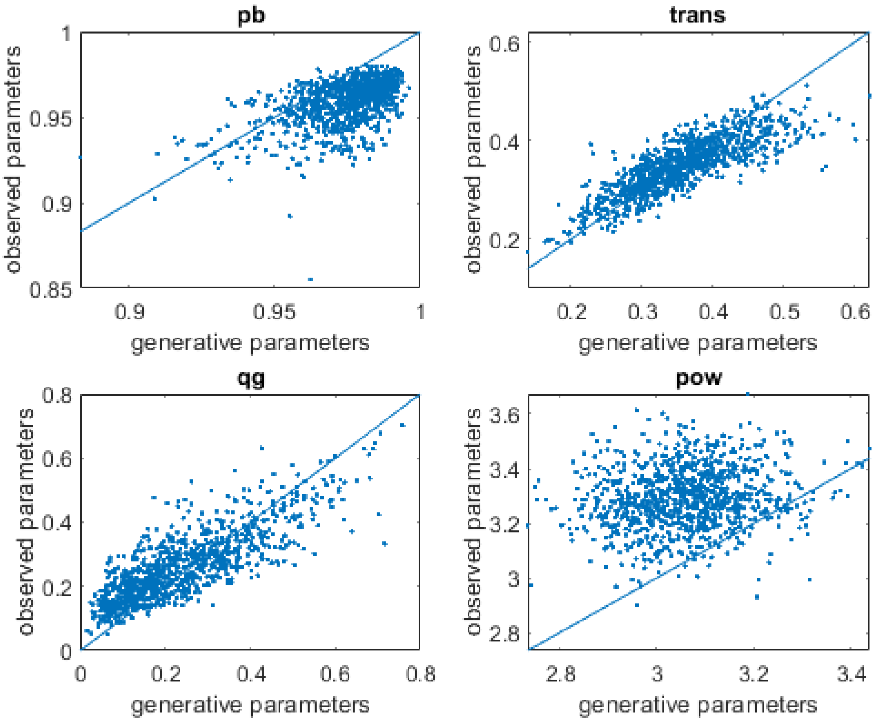

Figure S.5. Confusion matrix showing the model frequencies obtained for synthetic data generated with different model for the reversal task. Models: ba = beta-alpha; baf = beta-alpha-forget; bafc = beta-alpha-forget-conf; HMM3 = hidden Markov model with 3 free parameters; HMM4 = hidden Markov model with 4 free parameters.

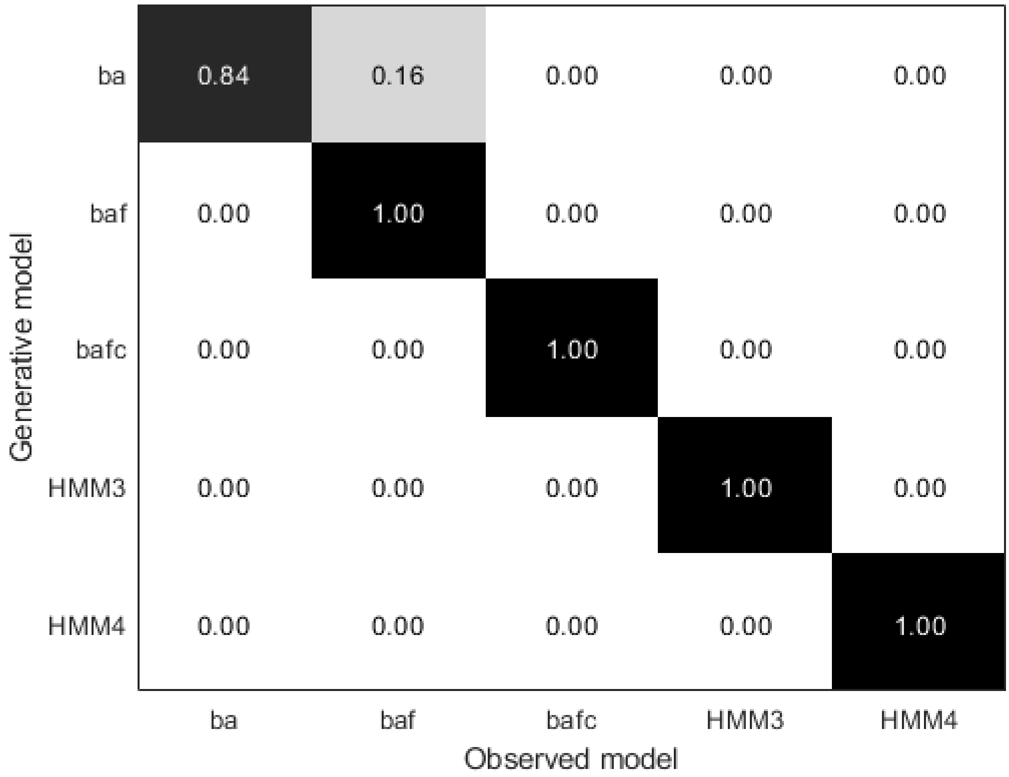

Supplement: Supplementary file 1 — Supplementary Information. [file 41598_2023_45179_MOESM1_ESM.pdf]
